# Biotin-Decorated Inulin-Based Polymeric Micelles Unveil Their Dual-Targeting Ability for the Potential Treatment of Glioblastoma Multiforme through the *In Vitro* and *In Vivo* Investigations

**DOI:** 10.1021/acs.molpharmaceut.5c01861

**Published:** 2026-04-13

**Authors:** Paola Riccobelli, Elena Cannone, Serena Filiberti, Giovanni Ribaudo, Sara Anna Bonini, Antonella Grigoletto, Maria Luisa Massardi, Silvia Codenotti, Marco Schiavone, Roberto Ronca, Delia Mandracchia

**Affiliations:** † Department of Molecular and Translational Medicine, 9297 University of Brescia, Viale Europa 11, Brescia 25123, Italy; ‡ Department of Pharmaceutical and Pharmacological Sciences, 165490 University of Padova, Via Marzolo 5, Padova 35131, Italy

**Keywords:** dual-targeting, glioblastoma multiforme, biotin, inulin-vitamin E, micelles, curcumin, zebrafish, flow cytometry

## Abstract

This study evaluated biotinylated-inulin vitamin E micelles (INVITE-BIO) as a carrier for the hydrophobic drug curcumin and their biotin-mediated dual-targeting ability to the Blood–Brain Barrier (BBB) and Glioblastoma Multiforme (GBM) cells by *in vitro* and *in vivo* studies. We previously demonstrated that INVITE-BIO micelles are long-circulating carriers upon i.v. administration, remaining in the body for up to 48 h, making these nanosystems potentially useful for receptor-mediated targeted drug delivery. Here, we first report the physicochemical characterization of curcumin-loaded INVITE-BIO micelles, which revealed a nanosized and spherical shape, as evaluated by DLS and TEM. Moreover, INVITE-BIO micelles showed high loading capacity and good ability to release the payloaded curcumin, which is located in the core of micelles, as demonstrated by ^1^H NMR study. The bioavailability of biotin on the micelle’s surface was demonstrated by HABA/avidin binding assay. Second, *in vitro* biological studies on GBM U87MG cells indicated that empty micellar carriers did not reveal any significant cytotoxicity and that the encapsulation of curcumin within the carrier can enhance the curcumin’s efficacy, potentially by improving its bioavailability. Furthermore, confocal microscopy and flow cytometry studies revealed that the presence of biotin moieties is pivotal to enhancing the cellular uptake and retention of the INVITE nanosystems. In conclusion, INVITE-BIO micelles enhanced both the crossing of BBB and drug accumulation in GBM tumor cells overexpressing the receptor for biotin through receptor-mediated endocytosis, demonstrating the great potential of biotinylated INVITE micelles as a promising dual-targeted approach for i.v. administration of antitumoral drugs for the treatment of Glioblastoma Multiforme.

## Graphical abstract

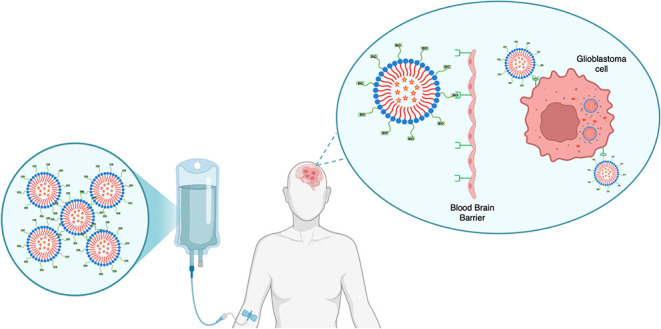

## 1 Introduction

Glioblastoma multiforme (GBM) is one of the most aggressive and malignant brain tumors in adults, and it is classified as a grade IV astrocytoma by the World Health Organization (WHO) classification. Its incidence is higher in men, and prevalence increases with age, peaking between 45 and 70 years.^1^ GBM is characterized by rapid progression, poor prognosis, and a median survival of 12–18 months postdiagnosis, with only 8.7% of patients surviving beyond 2 years.^2,3^

The treatment of GBM is exceptionally challenging due to its highly infiltrative nature, tumor heterogeneity, immunosuppressive microenvironment, multiple dysregulated signaling pathways, and mutations.^2,4^

The current standard treatment includes maximal surgical resection, followed by radiotherapy and chemotherapy with the gold standard Temozolomide (TMZ). However, despite their potential, the efficacy of these therapies is limited by several factors, including the inability to completely remove the tumor, the difficulty in crossing the Blood–Brain Barrier (BBB), and the lack of specific targets that would allow drugs to reach the tumor site without requiring high doses. These challenges underscore the growing need to develop and propose new approaches for treating glioblastoma.^5^

Among all the proposed new therapies, nanosystems seem to be very attractive because of their small size (from 10 to 100 nm) and their ability to encapsulate different drugs, improving their solubility and protecting them from the attacks of the immune system and from the degradation of the surrounding environment. Moreover, nanoparticles are promising materials for the dual-targeting approaches in the cancer therapies, especially for the selective targeting of the Central Nervous System (CNS).^6^ In fact, nanoparticles can be functionalized with specific ligands, enabling them to overcome the BBB and, at the same time, to reach the GBM cells.^4,7^

In particular, with respect to the GBM treatment, the dual-targeting strategy refers to the possibility of using one or more target ligands able to transport the system beyond the BBB and then direct it to the GBM cells (Scheme 1). This can lead to different potential benefits, such as the reduction of drug resistance due to the protective effect of the nanosystems toward loaded drugs from the attack by the immune systems and from the activity of the efflux pumps; a higher chance of therapy success by directing the system toward the site of action; and, in addition, a significant cost reduction due to the use of only one system to convey drugs through the BBB and directly to the GBM cells.^8,9^

**Scheme 1:**
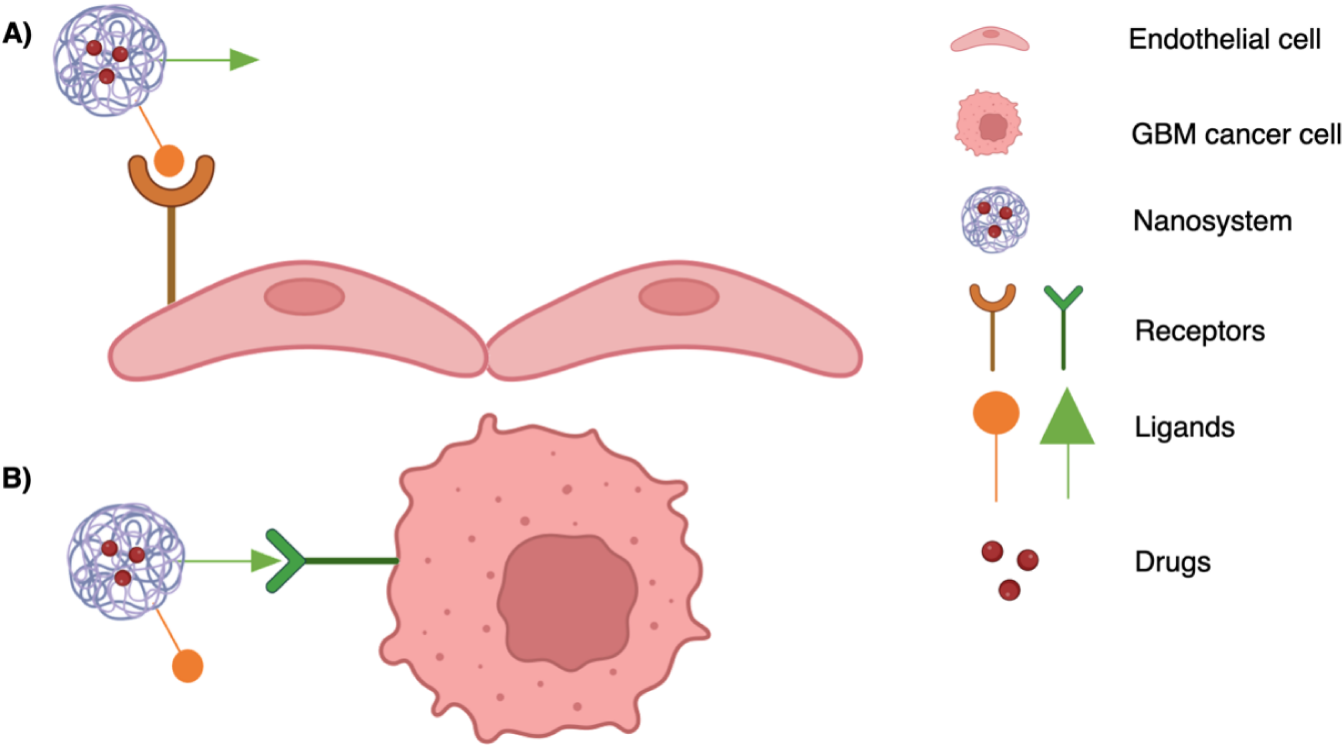
Schematic Representation of a Generic Functionalized Dual-Targeting Nanosystem (DTN) Containing Two Different Ligand Molecules That Make It Able to: (A) Cross the BBB via RMT; (B) Selectively Reach the GBM Cells by Interacting with Receptors Overexpressed on Cancer Cell Surfaces

In this context, Biotin (BIO), also known as vitamin B8, seems to be one of the most promising targeting agents. It is well-known that the uptake of biotin by mammalian cells is receptor-mediated, and its main transporter has been found in the sodium-dependent multivitamin transporter (SMVT), overexpressed in several aggressive cancer lines such as leukemia (L1210FR), ovarian (OV 2008, ID8), colon (Colo-26), mastocytoma (P815), lung (M109), renal (RENCA, RD0995), and breast (4T1, JC, MMT06056) cancer cell lines.^10^

On the other hand, studies evidenced that biotin is, moreover, a promising ligand for dual-targeting the CNS and, in particular, GBM cells because its surface receptors, the monocarboxylate transporters (MCTs), are overexpressed on both the BBB and GBM cells.^11,12^ Moreover, biotin also binds to the SMVT, which is expressed on the Blood–Brain Barrier (BBB), thus enabling a dual-targeting strategy that facilitates drug transport both to GBM cells and across the BBB.^13,14^

Over the years, researchers developed different biotinylated nanosystems with the aim of efficiently targeting the brain. For example, Uram et al. in 2019 developed Generation 3 biotinylated PAMAM dendrimers (G3-BCL) substituted with celecoxib, a COX-2 inhibitor with anticancer properties, and Fmoc-l-Leucine, a Peroxisome Proliferator-Activated Receptor (PPARγ) agonist that enhances cancer treatment efficacy. Here, biotin enhances drug transport across the BBB via binding to overexpressed receptors, the SMVT and MCT-1 and MCT-8. These systems effectively were able to cross the BBB and to release anticancer agents to brain tumors.^13^ Neves et al. developed Solid Lipid Nanoparticles (SLNs) functionalized with Apolipoprotein E (Apo E) and BIO. Apo E was able to target Low-Density Lipoprotein receptors (LDL receptors), abundant on the BBB, and the increased biotin levels in tumor tissue enhance specificity for cancer cells. The results demonstrated an improved brain permeability and enhanced drug delivery through the BBB. Furthermore, a higher uptake and permeability were observed with biotinylated solid lipid nanoparticles compared to nonbiotinylated SLNs.^15–17^

These studies furthermore highlight the need to optimize drug delivery to GBM cells, for example, by developing a dual-targeting system able to modulate the drug pharmacokinetic profile, actively cross the BBB, and enhance cellular uptake by GBM cells.

The present work aimed to evaluate the potential of INVITE-BIO, biotinylated polymeric micelles composed of natural molecules inulin (INU) and vitamin E (VITE), as dual-targeting systems for crossing the BBB and selectively delivering hydrophobic drugs to GBM cells.

In our previous studies, we reported on Inulin and Vitamin E polymeric micelles (INVITE) that have shown promising characteristics for their application in anticancer therapy, including: 1) the ability to load and release hydrophobic drugs in a controlled way;^18,19^ 2) the ability to cross biological membranes;^20^ 3) long-circulation characteristics after i.v. administration;^19,21^ and 4) antiangiogenic activity.^22^

In addition, biotin-decorated INVITE micelles (INVITE-BIO) showed long-circulating behavior useful for receptor-mediated targeted drug delivery, and *in vitro* studies on Caco-2 cell monolayers indicated that the transport of INVITE-BIO micelles was faster than that of surface-unmodified INVITE micelles.^23^

Moreover, *in vivo* optical imaging studies evidenced that, upon intravenous administration, INVITE-BIO micelles were quantitatively present in the body up to 48 h.^23^

Given our previous findings and considering biotin’s potential as a dual-targeting delivery agent to the CNS, this study investigates the ability of the long-circulating biotin-surface-modified polymeric INVITE micelles to act as dual-targeting drug delivery carriers for the potential treatment of glioblastoma multiforme (GBM) by enhancing BBB crossing and drug accumulation in GBM tumor cells overexpressing the biotin receptors.

Curcumin was chosen as a hydrophobic fluorescent model drug, and in addition, it is reported in recent literature as a potential candidate for GBM treatment.

In fact, curcumin acts on multiple cellular pathways that are crucial for glioblastoma progression. It inhibits the Janus Kinase/signal transducers and activators of transcription (JAK/STAT) pathway, specifically disrupting STAT3 signaling linked to tumor growth, migration, and epithelial-mesenchymal transition (EMT), a process crucial for metastasis.^24^ CUR also targets the phosphoinositide 3 kinase (PI3K)/Akt/mammalian target of the rapamycin (PI3K/Akt/mTOR) pathway, suppressing Akt and mTOR phosphorylation to inhibit GBM cell proliferation and migration. Additionally, CUR affects the Wnt/β-catenin pathway by reducing the β-catenin concentration and Hepatoma-Derived Growth Factor (HDGF) activity, further reducing GBM cell proliferation and migration. Other key pathways influenced by curcumin include Tumoral protein 53 (p53) and RB, Mitogen-Activated Protein Kinase (MAPK), Nuclear Factor Kappa-light-chain-enhancer of activated B cells (NF-κB), and Sonic Hedgehog gene (Shh), which control tumor growth and angiogenesis.^25^

For example, Hesari et al. showed that curcumin-loaded nanomicelles inhibited GBM cell growth, proliferation, and invasion by modulating the Wnt/β-catenin and NF-κB pathways.^26^

Furthermore, Walker and Mittal demonstrated that CUR inhibits angiogenesis by reducing tumor blood vessel formation and decreasing proangiogenic factors such as Vascular Endothelial Growth Factor (VEGF) and basic Fibroblast Growth Factor (bFGF). In addition, it also limits cell invasion and migration by reducing the expression of several matrix metalloproteinases (MMPs) (i.e., MMP-2, MMP-9, MMP-14, etc.). Curcumin can moreover generate Reactive Oxygen Species (ROS), reducing GBM cell viability and sensitizing tumors to radiation therapy, increasing selective cytotoxicity.^25^ These findings support the potential of curcumin-loaded nanomicelles as promising therapeutic agents for GBM.

Although curcumin was chosen in this study due to its hydrophobic nature and relevance as an anticancer agent, the INVITEBIO nanomicellar platform could, in principle, encapsulate and deliver other small, lipophilic drugs with physicochemical properties similar to those of curcumin, such as comparable molecular weight and lipophilicity. Nitrosoureas like carmustine (BCNU) and lomustine (CCNU), as well as hydrophobic polyphenols such as resveratrol or quercetin, represent examples of compounds whose characteristics make them compatible with this micellar system, supporting the versatility of INVITEBIO for a broader range of hydrophobic agents for GBM therapy.^27,28^

In this work, biotinylated INVITE micelles loaded with curcumin (CUR), named INVITE-BIO/CUR, were developed and investigated as potential dual-target delivery systems. The presence of biotin on the micelle surface was evaluated for active targeting purposes. *In vitro* studies on U87MG glioblastoma cells and *in vivo* biodistribution studies in zebrafish were performed to assess the ability of these micelles to cross the BBB and promote cellular uptake in GBM cells. Overall, this work proposes INVITE-BIO/CUR micelles as promising dual-targeting nanocarriers for GBM drug delivery.

## 2 Experimental Section

### 2.1 Chemicals

All reagents were of analytical grade unless otherwise stated.

Triethylamine ≥99% (TEA), *N*,*N*′-dicyclohexyl carbodiimide 99% (DCC), inulin from dahlia tubers (INU, approximately 5500 Da), *N*-hydroxysuccinimide (NHS), D-α-tocopherol succinate (Vitamin E succinate, VITE), curcumin (CUR), Tween 80, and HABA/Avidin reagent were purchased from Merck (Milan, Italy). *N*-hydroxysulfosuccinimide sodium salt ≥98% (NHSS) and biotin (BIO) were purchased from TCI Europe (Zwijndrecht, Belgium). Dimethyl sulfoxide (DMSO) and anhydrous *N*,*N*-dimethylformamide 99.8% (DMF) were purchased from Carlo Erba Reagents (Milano, Italy). Diethyl ether, acetone, NaCl, Na_2_HPO_4_, KH_2_PO_4_, and DMSO-*d*_6_ were purchased from VWR (Milano, Italy). Dialysis tubes with a MWCO of 3500 Da (Spectra/Por 6) were purchased from Spectrum Laboratories.

### 2.2 Cell Line

The human malignant glioblastoma U87MG cell line (HTB-14) was cultured in a Dulbecco’s modified Eagle’s medium (D-MEM, Sigma-Aldrich) with 4.5 g/L glucose supplemented with 10% Fetal Bovine Serum (FBS, Sigma-Aldrich), 50 mg/mL penicillin, and 100 mg/mL streptomycin (Sigma-Aldrich). Cells were grown at 37 °C in a 95% air–5% CO_2_ humidified incubator.

### 2.3 Zebrafish Maintenance

AB Zebrafish wild-type (WT) have been maintained at 28.5 °C, exposed to a 14–10 light–dark cycle in aerated saline water, in the Zebrafish Facility at the University of Brescia, according to standard protocols suggested by Animal Care Standard Operating Procedures (ARRIVE guidelines 2.0) and the Italian Ministry of Health. To allow animal breeding, resulting in egg deposition and fertilization, animals have been sorted by sex in the late afternoon and freed the next morning to allow mating events. Embryos have been collected and maintained at 28.5 °C in embryo medium (0.19 g/L CaSO_4_, 0.1 g/L Instant Ocean Salt and 0.1 g/L NaHCO_3_). Manipulation and experiments have been carried out before 120 hpf; therefore, for instance, no additional ministerial authorization is needed.

For each experiment involving zebrafish samples, pools of at least 5–10 embryos were treated, and all experiments were repeated in triplicate.

### 2.4 Apparatus

UV–vis analyses were performed by using a Jasco V-530 UV/vis Spectrophotometer (Cremella, Italy).

^1^H NMR spectra were recorded using a Bruker Ascend spectrometer (frequency: 400.13 MHz for ^1^H) (Bruker, Billerica, MA).

Centrifugations were performed with a Megafuge 1.0 R, Heraeus Instruments (Germany), equipped with temperature control.

Lyophilization was performed by a FreeZone freeze-dryer, LABCONCO (Milan, Italy), at −50 °C and 0.1 mbar.

Size measurements were conducted by a Zetasizer NanoZS (Malvern Instruments Ltd., Worcestershire, UK).

TEM analyses were performed by using a Jeol-JEM-1200EXII microscope (Japan).

The synthesis of bioconjugates and the release studies were conducted by using an SKI4 shaking incubator, ArgoLab (Modena, Italy).

Cellular uptake studies of curcumin-loaded micelles were conducted with a ZEISS LSM 900 confocal laser-scanning microscope (Carl Zeiss, Germany).

Cytofluorimetric analyses of curcumin-loaded micelles were conducted using a MACSQuant Analyzer (Miltenyi Biotec, Bergisch Gladbach, Germany).

### 2.5 Synthesis and Characterization of INVITE and INVITE-BIO Conjugates

The INU-VITE bioconjugate (INVITE) was synthesized as previously reported.^18^ Briefly, the carboxyl group of VITE succinate (1 equiv) was dissolved and activated in anhydrous DMF under nitrogen with DCC (2 equiv) and NHSS (2 equiv) under stirring for 3 h at 25 °C. The amount of VITE was added according to the following molar ratio: *Y* = 0.2, where *Y* indicates the molar ratio VITE/INU-repeating-units.

Then, in this mixture, TEA, as a catalyst, was added according to the following molar ratios: *Z* = 0.10, where *Z* indicates the molar ratio TEA/INU-repeating-units.

The reaction was carried out under nitrogen at 25 °C for 24 h. After this time, the solution was centrifuged to remove the formed dicyclohexylurea, and the resulting solution was precipitated in acetone. In this solvent, inulin is insoluble, while DCC, NHSS, and VITE are freely soluble. So, the precipitation solvent was also used for the purification of the final products.

The synthesis of INVITE-BIO, a biotin-decorated INVITE conjugate, is based on the isolation of the *N*-hydroxysuccinimide ester of BIO, as previously reported.^23^

Briefly, INVITE was dissolved in anhydrous DMF, and the INVITE polymer, in the presence of TEA, freely and quantitatively reacted with the synthesized BIO-NHS. The added amount of TEA was chosen according to the following molar ratio *Z* = 0.5, where *Z* indicates the molar ratio TEA/INU-repeating-units, while the amount of BIO-NHS was added according to the following molar ratio *Y* = 0.25, where *Y* indicates the molar ratio BIO-NHS/INU-repeating-units.

The reaction was carried out under nitrogen at 25 °C for 24 h. After this time, the solution was precipitated in diethyl ether and washed with the same solvent several times to remove impurities.

The obtained powders of INVITE and INVITE-BIO were dried under a vacuum.

Then ^1^H NMR spectra of INVITE and INVITE-BIO were acquired after exhaustive dialysis to ensure high purity of the samples, and the data were in agreement with previous results.^18,23^

### 2.6 Preparation of Empty or Curcumin-Loaded INVITE and INVITE-BIO Micelles

Empty micelles of INVITE and INVITE-BIO or loaded ones with CUR called INVITE/CUR and INVITE-BIO/CUR were prepared by the dialysis method. To ensure the CUR loading within the micelles, the polymer concentration was always maintained above their Critical Association Concentration (CAC), previously determined (Figure S1).^22,23^ Calculated amounts of INVITE and INVITE-BIO were dissolved in dimethyl sulfoxide with or without CUR (5% (w/w)) with respect to the polymer and left under constant stirring for 1 h. The resulting solution was poured in a dialysis tube (Spectra/Por 6) with a MWCO of 3500 Da, dialyzed against distilled water for 3 days, and lyophilized. The resulting solution was transparent for INVITE and INVITE-BIO and yellow when there was CUR loaded in the micelles. The obtained solutions were dried and then lyophilized at −50 °C and 0.1 mbar for 4 days.

### 2.7 Evaluation of Drug Loading and Encapsulation Efficiency on Curcumin-Loaded INVITE and INVITE-BIO Micelles

For the evaluation of Drug Loading (DL), 4 mg of lyophilized INVITE/CUR and INVITE-BIO/CUR micelles were dissolved in 10 mL of DMSO and left under constant stirring for 3 h. Then, the solutions were diluted 1:5 with the same solvent, and the amount of loaded CUR was determined by UV–Vis spectrophotometry by reading at 425 nm. Each measurement was performed in triplicate. The loaded amount of CUR was calculated by a calibration curve of CUR in DMSO in the concentration range of 4·10^–3^ to 5·10^–4^ mg/mL (correlation coefficient R^2^ > 0.999).

The drug loading (DL) and the encapsulation efficiency (EE) of CUR micelles were calculated according to the eqs 1 and 2 ^29^

1 DL%⁡(w/w)=(CUR in micelles)/(micelles+CUR)×100

2 EE%⁡(w/w)=(experimental drug loading/amount of drug used in the loading experiment)×100

### 2.8 Morphological Characterization of the INVITE/CUR and INVITE-BIO/CUR Micelles

The morphological characteristics of INVITE/CUR and INVITE-BIO/CUR micelles were further examined by Transmission Electron Microscope (TEM). In particular, a drop of micelle solution (1 mg/mL) was deposited on a copper grid, left drying at 25 °C in a desiccator, and then negatively stained with uranyl acetate and analyzed by TEM.

### 2.9 Quantification of Biotin on INVITE-BIO Micelles

The amount of available biotin on micelles was determined by the HABA/avidin binding assay. The HABA/avidin reagent was reconstituted with 10 mL of deionized water, according to the Sigma-Aldrich datasheet.

The UV absorbance of 900 μL of HABA/avidin reagent solution at λ = 500 nm was measured. Then, 100 μL of sample was added to this solution and mixed by inversion, and the absorbance at λ = 500 nm was read.

The amount of the available biotin, which corresponds to the μmoles of biotin per milliliter of the sample solution, was calculated by the following formula:^30^

$$\mu mol\;biotin/mL=\left(\Delta A_{500}/34\right)\times 10$$

where ΔA_500_ = 0.9 (A_500_ HABA/avidin) – (A_500_ HABA/avidin + sample).

### 2.10 Size Measurements and Stability Studies on Empty and Curcumin-Loaded INVITE-BIO Micelles

INVITE-BIO micelles, empty or loaded with CUR, were prepared by the above-described direct dialysis method. The hydrodynamic size of the INVITE-BIO and INVITE-BIO/CUR micelles and the polydispersity index were measured by using the Zetasizer Nano ZS instrument.

In particular, lyophilized micelles were dispersed in ultrapure water at a concentration of 0.5 mg/mL, left to equilibrate for 3 h at 25 °C under gentle stirring, followed by filtration through 0.45 μm nylon membranes to remove aggregates. The solutions were further kept at 25 °C for a predefined storage time of up to 6 days to verify their physical stability, evaluated by measuring the size of the micelles after these periods of storage. All measurements were carried out in triplicate and reported as the mean ± SD.

### 2.11 Drug Release Studies from INVITE-BIO Micelles Loaded with CUR

*In vitro* release studies were performed by the dialysis method.^22^ In particular, 10 mg of INVITE-BIO/CUR were solubilized in 3 mL of double-distilled water, and the solutions were poured into a 3500 Da MWCO dialysis membrane. The dialysis tube was incubated in 10 mL of PBS at pH 7.4 or PBS at pH 5.5 containing polysorbate 80 (3 wt %) at 37 °C under stirring (100 rpm).

At established release points (30 min, 2 h, 4 h, 6 h, 24 h, 48 h), the entire release medium was removed and replaced by prewarmed fresh release media. The amount of released CUR has been determined by UV–Vis spectrophotometry at 425 nm by comparing the data with a calibration curve of CUR measured in the same medium in the concentration range of 3·10^–4^ to 1·10^–1^ mg/mL (correlation coefficient R^2^ > 0.999). All analyses were carried out in triplicate.

### 2.12 *In Vitro* Antiproliferative Activity of Curcumin-Loaded Micelles

U87MG cells were seeded (2 × 10^4^) in 48-well culture plates in complete medium. After 24 h, the cells were treated with curcumin-loaded micelles or control in medium with 1% FBS. Cells were subsequently harvested at 72 h and counted using the MACSQuant system.

### 2.13 *In Vitro* Cellular Internalization Studies by Confocal Microscopy

To perform the immunofluorescence assay, U87MG cells were plated with a density of 180000 cells/well in a 24-well plate and grown on a glass coverslip (coated with poly l-lysine, Sigma-Aldrich). The cells were treated the day after seeding, to give them time to adhere to the slide, with the following treatments: control (treatment with vehicle, DMSO 1:1000), curcumin (10 μg/mL), curcumin-loaded INVITE micelles (INVITE/CUR), and curcumin-loaded INVITE-BIO micelles (INVITE-BIO/CUR).

After 4 h, cells were fixed in ice-cold methanol (Sigma-Aldrich), then washed and incubated in Phosphate Buffered Saline (PBS, Sigma-Aldrich) containing 1% of Bovine Serum Albumin (BSA, Sigma-Aldrich) and 0.2% Triton X-100 overnight at 4 °C with a monoclonal anti-actin (Sigma-Aldrich, 1:600). After rinses, cells were incubated with CYTM3-conjugated antimouse secondary antibody (Jackson ImmunoResearch Laboratories Inc., 1:1000) in PBS for 1 h at room temperature. ToPro3 staining (5 min incubation, 1:1000, Thermo Fisher Scientific) was also used for nuclei visualization. Curcumin naturally absorbs light in the blue light wavelengths (maximum absorption peak ∼408–430 nm) and emits light in the range of 460–560 nm.^31^ To evaluate curcumin internalization into cells, slices were mounted and examined by a ZEISS LSM 900 confocal laser-scanning microscope (Carl Zeiss, Germany). Images were processed using ImageJ software (National Institutes of Health).

The curcumin fluorescence intensity was determined by analyzing 10 fields for each treatment. In order not to create disparities between fields with different cellular densities, 10 areas/field were evaluated, and fluorescence was measured in circular areas with a diameter of 20 μm, corresponding approximately to the size of a cell body.

### 2.14 *In Vitro* Cellular Uptake and Retention Studies by Flow Cytometry Analysis

U87MG cells were seeded (8 × 10^4^) in 48-well culture plates in complete medium. After 24 h, the cells were treated with curcumin-loaded micelles or control in medium with 1% FBS. Cells were subsequently harvested at different time points. Fluorescence analysis was performed using excitation with a VioBlue-A laser on the MACSQuant system.

### 2.15 *In Vitro* Receptor Competition Assay

U87MG cells were seeded (8 × 10^4^) in 48-well culture plates in complete medium. After 24 h, the cells were treated with curcumin-loaded micelles or control in medium with 1% FBS, in the presence or absence of a 10-fold excess of free biotin. Cells were subsequently harvested at 3 h after treatment. Fluorescence analysis was performed using excitation with a VioBlue-A laser on the MACSQuant system.

### 2.16 *In Vivo* Biological Studies on Zebrafish

For teratogenicity evaluation purposes, free CUR, INVITE/CUR, and INVITE-BIO/CUR at the concentration of 10 μM were dissolved in embryo medium and used to treat zebrafish eggs via immersion at the 2-cell stage, after nonfertilized or degenerated eggs removal. Embryos were examined every 24 h until reaching 2 days post fertilization (dpf).

For fluorescence quantification experiments, embryos were dechorionated at 48 hpf and injected with 4 nL of a 2 mM solution of free CUR, INVITE/CUR, and INVITE-BIO/CUR into the duct of Couvier. The injected volume of 4 nL was selected based on previous data demonstrating that injections of up to 5 nL into the bloodstream are safe in 48 hpf zebrafish. Since the estimated blood volume ranges between 50 and 90 nL, a 4 nL injection does not exceed 10% of the total zebrafish blood volume at 48 hpf.^32,33^ To have an adequate control, untreated WT were always kept in the same conditions, and for injection experiments, controls were injected with sterile water. Images were taken with the Axiozoom V16 equipped with Axiocam. After a brief anesthesia with tricaine, samples were embedded in 2% methylcellulose and positioned with a lateral orientation.

### 2.17 Statistics

Statistical analyses were performed using GraphPad Prism 8. Fluorescent quantification has been performed with a one-way ANOVA test with Bonferroni correction, and data have been reported as mean ± standard error of the mean. In all of the analyses, the statistical significance was fixed at *p* ≤ 0.05.

### 2.18 Ethical Use of Animals or Human Participants in Research

All zebrafish embryo (*Danio rerio*) experiments were conducted in accordance with institutional guidelines and European Directive 2010/63/EU for animal experiments. Zebrafish embryos were used before 5 days post-fertilization and are therefore not considered protected animals; consequently, ethical approval was not required.

## 3 Results and Discussion

Delivering drugs directly to the tumor site has long been a challenge for pharmaceutical technologists. In the literature, a large number of articles have dealt with this problem.

In particular, delivering therapeutics to brain tumors via systemic circulation is further complicated by the presence of the BBB.

Among the proposed strategies, the incorporation of drugs into properly functionalized nanosystems seems to be one of the most suitable methods, and especially the dual-targeting strategy can be a useful method to allow nanosystems to overcome the BBB and reach the cellular target.

In this work, we show, for the first time, that biotinylated inulin-D-α-tocopherol micelles (INVITE-BIO) exhibit a dual-targeting ability. This is demonstrated by an *in vivo* biodistribution study using live imaging to assess BBB passage in a zebrafish model and by the selective targeting to the GBM cells, evidenced by increased internalization and retention. Consequently, INVITE-BIO nanomicelles represent a potentially useful system for the treatment of Glioblastoma Multiforme.

The INVITE micelles with a hydrophobic core of vitamin E and a hydrophilic shell of inulin^21^ were chosen as the base vector. This choice was driven by our previous studies, which have demonstrated the ability of INVITE micelles to load several hydrophobic drugs (i.e., curcumin, celecoxib, rifampicin), a long circulation after i.v. administration,^21^ and antiangiogenic activity,^22^ which make the INVITE platform a very promising system in oncology.

On the other hand, to achieve a dual-targeting strategy for glioblastoma, it was thought to exploit the long-circulating biotinylated-INVITE micelles (INVITE-BIO) prepared according to our previously reported procedure,^23^ and here, they have been loaded with curcumin (INVITE-BIO/CUR) and characterized from a physicochemical and biological point of view by *in vitro* and *in vivo* studies on zebrafish.

This approach takes advantage of biotin as a ligand whose receptors are overexpressed in both the BBB and GBM and of curcumin, which is not only an appropriate hydrophobic model drug but also an interesting molecule capable of acting on some signaling pathways involved in glioblastoma.

The INVITE and INVITE-BIO conjugates (Scheme 2A) have been prepared by a published method, and they have been characterized by ^1^H NMR, and the obtained data fitt with previous results.^18^

**Scheme 2:**
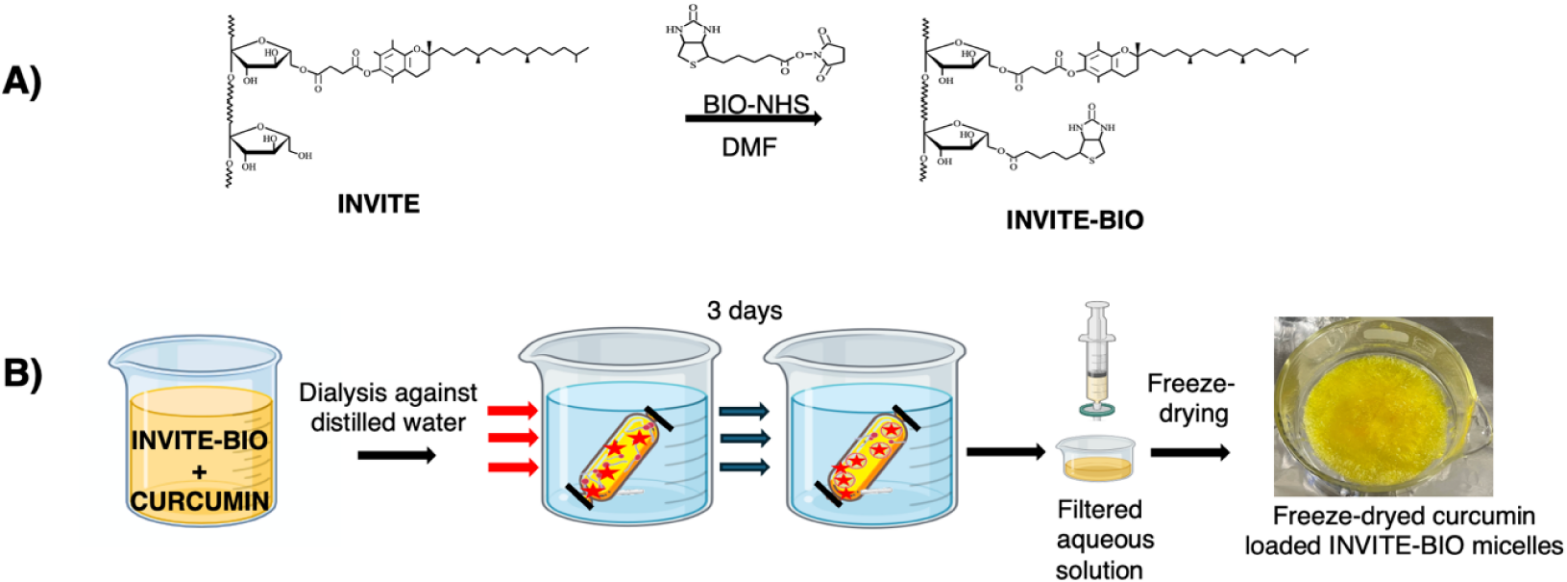
Schematic Illustration of: (A) the Synthesis of the INVITE-BIO Conjugate; and (B) the Preparation of CUR-Loaded INVITE-BIO Micelles via the Dialysis Method

Briefly, the ^1^H NMR spectrum of the INVITE conjugate was recorded in DMSO-d6 and confirmed the chemical conjugation between Inulin (INU) and Vitamin E (VITE) (Figure S2). The Derivatization Degree (DD% mol), calculated as the ratio between the integral of the signal at ∼0.80 ppm (m, 12H, 4 × CH_3_ alkyl chain VITE) belonging to VITE and the integral of the signals at 3.50–4.00 ppm belonging to the seven protons of the INU fructose ring, was 16.30% (mol/mol).

^1^H–NMR spectrum of the INVITE-BIO conjugate was recorded in DMSO-*d*_6_ and showed peaks at 0.86 ppm (12H VITE), 3.50–4.00 ppm (7H INU), 4.30 ppm (1H BIO), and 6.36–6.40 ppm (2NH BIO) (Figure S3). The peaks at ppm 4.30 (s, 1H, −NH–CH–CH–, ring BIO), 6.36 (s, 1H, NH, BIO), and 6.40 (s, 1H, NH, BIO) were used to identify BIO in the conjugate and to calculate the DD that was 16.00% (mol/mol) in agreement with the theoretical data.

Given the importance of biotin’s surface localization on micelles for effective interaction with its receptors, the bioavailability of biotin moieties on the surface of INVITE-BIO micelles was evaluated by a HABA/avidin binding assay. The binding of HABA to avidin gives an absorption maximum at 500 nm, and the absorbance will decrease when biotin or a biotinylated entity is added, which is due to the displacement of HABA from the complex with avidin by biotin (the K_d_ for the HABA/avidin complex is 6 × 10^–6^ at pH 4.7 vs that of approximately 10^–15^ M for the avidin–biotin complex). Moreover, the absorption decreases proportionately to the biotin present in the system, and the change in absorbance can be used to calculate the amount of biotin.^30^

The assay was conducted by measuring the absorbance of the avidin/HABA complex at 500 nm before and after being mixed with the INVITE-BIO micelles. Figure 1 shows the UV–visible spectra of the avidin/HABA complex before and after the addition of the INVITE-BIO micelles. As shown, upon addition of the biotin micelles, the absorbance of the avidin/HABA complex at 500 nm decreased, suggesting that micelles’ biotin displaced HABA from the avidin/HABA complex due to its higher affinity for avidin.

**Figure 1:**
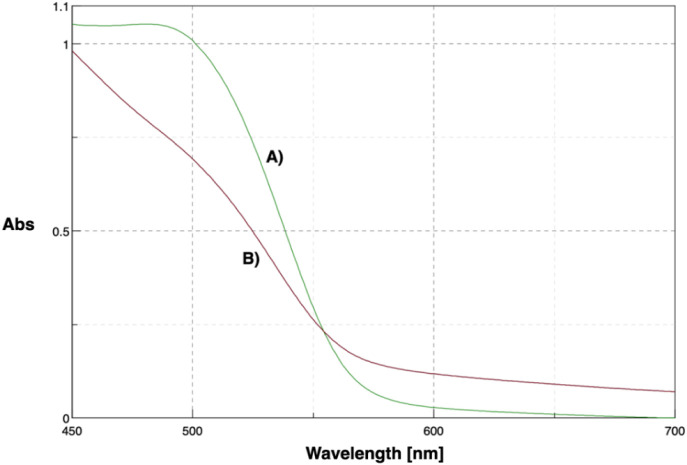
UV–vis spectra of: (A) HABA–avidin complex and (B) HABA/avidin complex + INVITE-BIO.

The amount of available biotin was calculated to be ca. 0.06314 μmol of biotin/ml, which corresponds to 15.4 μg/mg micelles. The bioavailability of biotin on the micelle’s surface represents an important feature for successful targeting toward overexpressed biotin receptors on the glioblastoma cell surface.

Aimed to evaluate the ability of INVITE-BIO to load the hydrophobic drug, CUR was chosen and loaded by dialysis (Scheme 2B). After comprehensive dialysis, the excess of CUR was removed by filtration (0.45 μm RC), and the samples were then lyophilized. The same procedure was used to load CUR into INVITE micelles (without biotin moieties).

^1^H NMR studies in D_2_O or DMSO-*d*_6_ have been performed to understand if CUR is located in the micellar core of INVITE-BIO, as stated for INVITE micelles by our previous studies.^34^

In D_2_O, the signals of CUR (6.5–7.5 ppm) disappear upon micelle formation (Figure 2), consistent with CUR being sequestered in the hydrophobic core, where restricted mobility and shielding attenuate the NMR signals. Such behavior has been widely used to probe drug partitioning in micellar systems.^35^ Probably, we can suppose that also in the case of INVITE-BIO micelles, the incorporation of CUR is determined by the π–π interactions of the benzopyran-6-yl residues of vitamin E and aromatic groups of curcumin, and the presence of biotin moieties does not interfere with the drug incorporation.^34^

**Figure 2:**
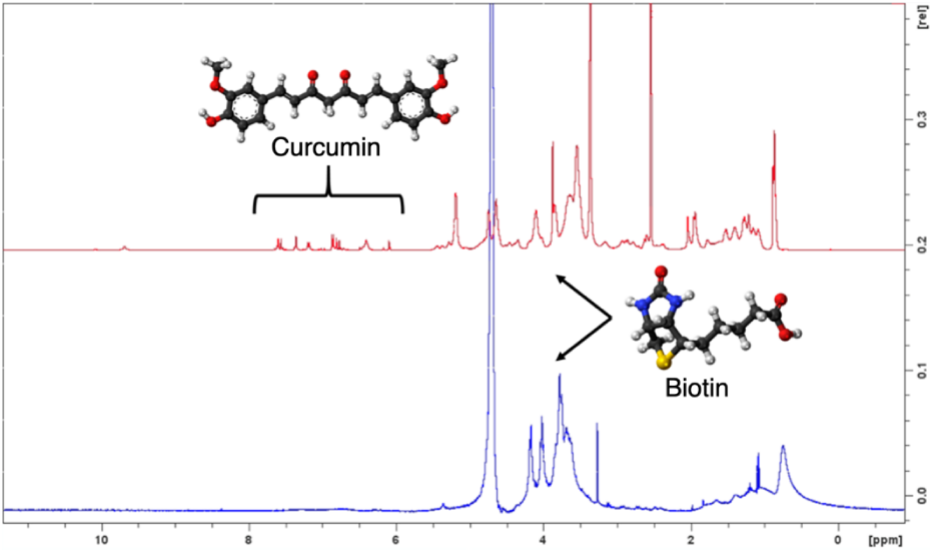
^1^H–NMR spectra of INVITE-BIO/CUR micelles recorded in DMSO-*d*_6_ (red spectrum) and D_2_O (blue spectrum).

To evaluate the drug loading (DL), INVITE-BIO micelles were dissolved in DMSO, and the total amount of loaded CUR was evaluated by UV–vis spectroscopy, and DL and Encapsulation Efficiency (EE) were calculated by the above-reported eqs 1 and 2.

The DL values reported in Table 1 show a good entrapment efficiency for INVITE-BIO with DL % = 3.1% w/w and EE % = 61.6% w/w.

**Table 1:** Physical-Chemical Behaviors of Empty and Curcumin-Loaded INVITE-BIO Micelles

Sample	Size (nm) ± sd	Đ^a^	DL % w/w	EE % w/w
INVITE-BIO	10.9 ± 0.5	0.35 ± 0.06	-	-
INVITE-BIO/CUR	11.8 ± 0.8	0.34 ± 0.05	3.1 ± 0.9	61.6 ± 4.2

a **Đ** = Dispersity.

Aimed at understanding the release behavior of the INVITE-BIO/CUR in physiologic fluids or in conditions simulating the tumor environments, release studies at pH 7.4 or 5.5 have been performed.

Figure 3 shows the drug-release profile, expressed as cumulative release percentage as a function of time in PBS solution at pH 7.4 or 5.5 in the presence of polysorbate 80 for up to 48 h.

**Figure 3:**
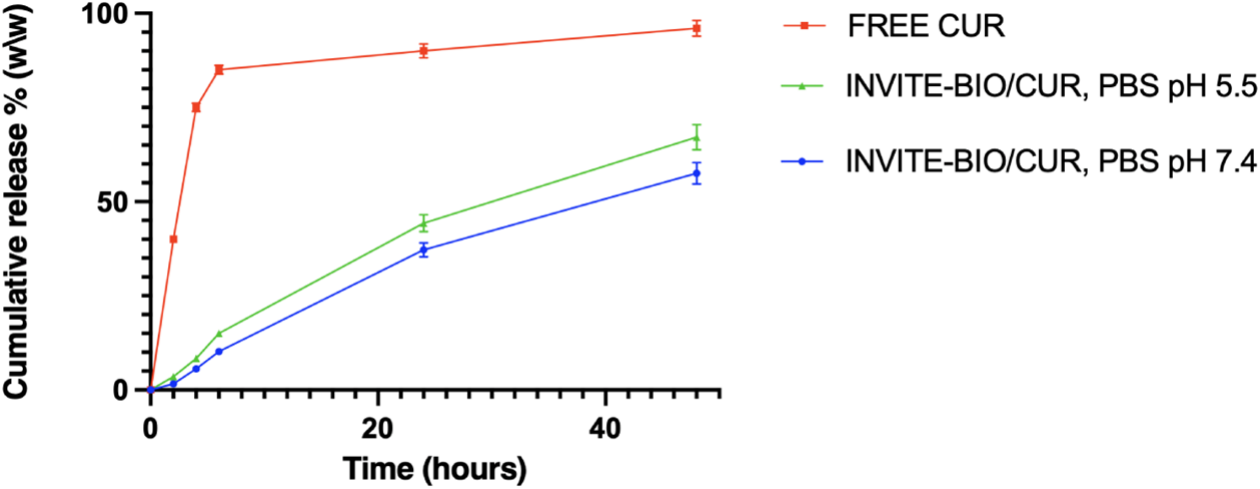
Cumulative release of curcumin from INVITE-BIO micelles at 37 °C in PBS at pH 7.4 or in PBS at pH 5.5.

During the release study, sink conditions were maintained throughout the experiment, and they were demonstrated by the dissolution curve of the free CUR that resulted in being quickly soluble in the release medium and were ensured by changing the whole release medium at each sampling time point.

As from Figure 3, the release curves from INVITE-BIO/CUR micelle systems clearly indicate a controlled release of curcumin in both media with no evident burst effect, reaching almost 60% of drug released at 48 h in PBS pH 7.4 and ∼70% at pH 5.5, ∼10% higher, which may be useful for tumor delivery in acidic environments. This release profile exhibited a similar rate of INVITE/CUR,^22^ suggesting that INVITE-BIO follows the same release mechanism and that the presence of biotin does not alter the micelle’s release behavior. On the other hand, the amount of CUR released from INVITE-BIO micelles was approximately twice that of the INVITE micelles. This difference can be attributed to the higher drug loading capacity of INVITE-BIO micelles, probably due to a possible curcumin-biotin interaction, which can influence the efficiency of drug encapsulation and, consequently, the overall release capacity.

The release profiles of INVITE-BIO micelles were analyzed according to common kinetic models (i.e., zero order, first order, Higuchi, and Korsmeyer and Peppas) but no statistically acceptable fits were found, suggesting a complex mechanism of drug release from this system.^22^

Furthermore, the micelles were characterized from a morphological point of view (Figure 4). Previous studies by SEM have shown that the INVITE, INVITE/CUR, and INVITE-BIO are round nanosystems around 7–20 nm.^20,22,23^ TEM studies have confirmed these data for INVITE/CUR (Figure 4A) and have shown similar results for INVITE-BIO/CUR micelles (Figure 4B), which are in agreement with the size obtained by DLS (Table 1). These results further confirm that curcumin is located at the level of the micelle core and does not cause a significant variation in their size.

**Figure 4:**
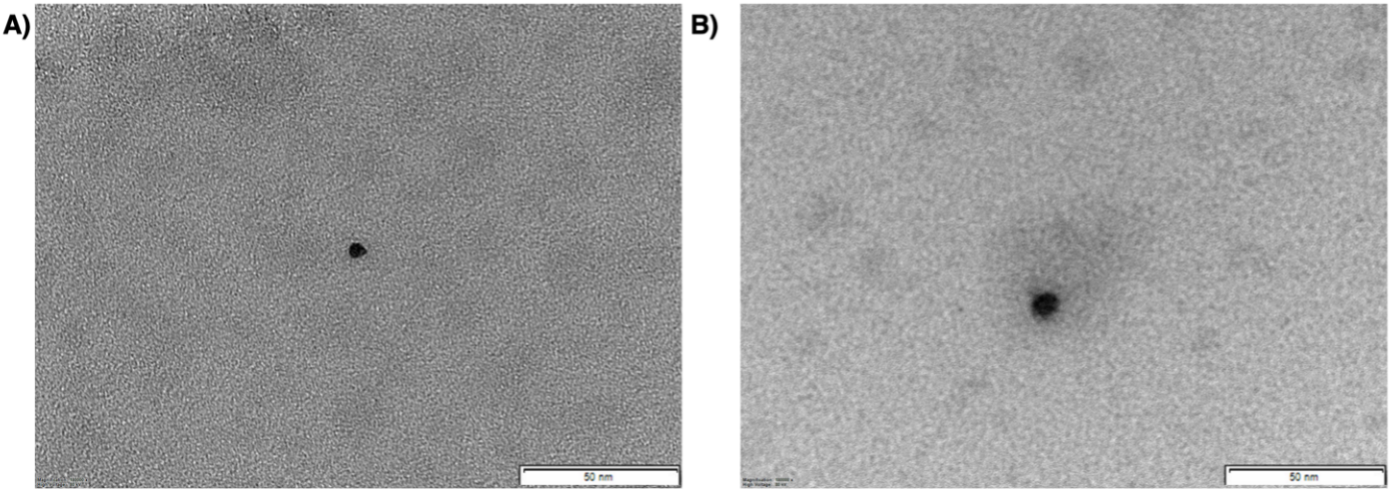
TEM images of INVITE/CUR (A) and INVITE-BIO/CUR (B) samples. The scale bar is 50 nm.

The nanometric size of INVITE-based micelles (naked or biotinylated), as determined by DLS and TEM, makes these systems very good candidates to freely cross the cell membrane and bring hydrophobic drugs like curcumin at the intracellular level. Indeed, several studies have demonstrated that nanosystems smaller than 20 nm can easily penetrate cell membranes via passive diffusion.^21,36^

For these reasons, it could be assumed that INVITE-BIO micelles can penetrate rapidly into the cell membrane, reaching high concentrations, as previously demonstrated for INVITE/CUR in a study of mesenchymal stromal cells.^20^

Biological *in vitro* studies were carried out to evaluate the effect of biotin functionalization of INVITE micelles on cellular proliferation, uptake, and retention in glioblastoma cells compared to nonbiotinylated ones.

Cellular proliferation studies of human glioblastoma cells (U87MG) showed that empty micelles display minimal cytotoxicity, with IC_50_ values of 35.92 and 43.35 μM for INVITE and INVITE-BIO, respectively (Figure 5B). Notably, these doses were higher than those of CUR-loaded micelles (Figure 5C), confirming their biocompatibility and that biotin functionalization does not significantly alter the micelle intrinsic antiproliferative effect under these *in vitro* conditions, so the observed effects on cellular proliferation are primarily due to the loaded drug.

**Figure 5:**
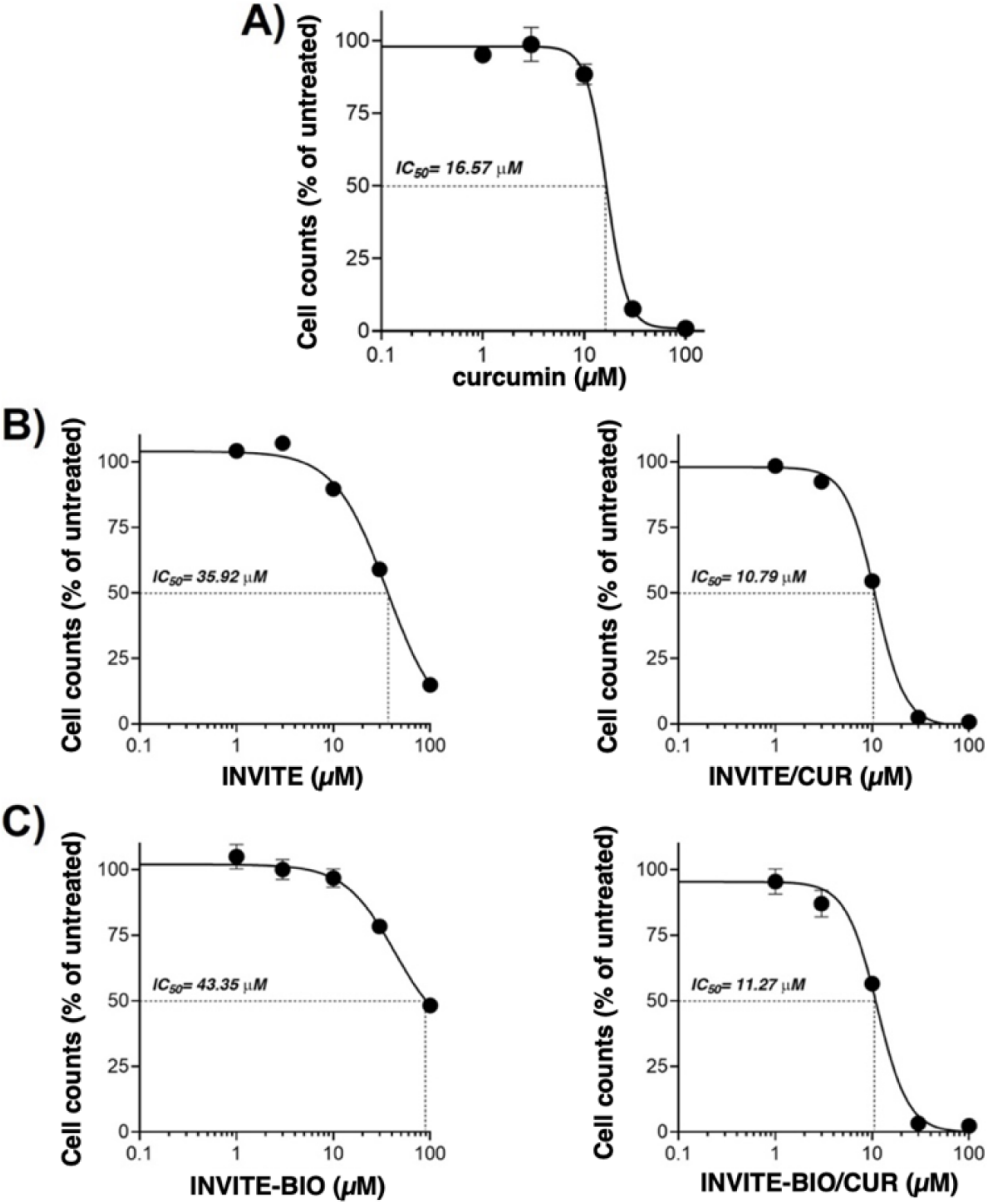
Cell proliferation of human glioblastoma cells (U87MG) treated for 4 h with (A) free curcumin; (B) empty INVITE (left) and INVITE-BIO (right) micelles; and (C) curcumin-loaded INVITE (left) and curcumin-loaded INVITE-BIO (right) micelles.

Moreover, both curcumin-loaded micelles, with and without biotin, exhibited improved antiproliferative activity compared to the free drug (Figure 5A) as indicated by lower IC_50_ values (10.79 and 11.27 μM for INVITE and INVITE-BIO, respectively, vs 16.57 μM of free curcumin), suggesting that the encapsulation of CUR within the carrier can enhance the curcumin’s efficacy, potentially by improving its bioavailability.

As postulated, the presence of biotin on the surface of INVITE micelles should allow a better interaction with the cell membrane overexpressing biotin receptors (like glioblastoma cells), leading to a more effective cellular internalization at the site of action.

To evaluate the internalization of curcumin-containing micelles in glioblastoma cells, microscopy studies were performed on the U87MG cell line treated with curcumin alone, INVITE/CUR micelles, and INVITE-BIO/CUR micelles. Microscopy studies showed that curcumin, due to its lipophilic characteristics, can, albeit to a minimal extent, enter cells. Its entry into glioblastoma cells, measured as green fluorescence intensity, was significantly increased when INVITE/CUR or INVITE-BIO/CUR micelles were used, and the greatest efficiency was achieved with INVITE-BIO micelles (Figure 6A). The fluorescence intensity was also quantified and reported in the graph in Figure 6B.

**Figure 6:**
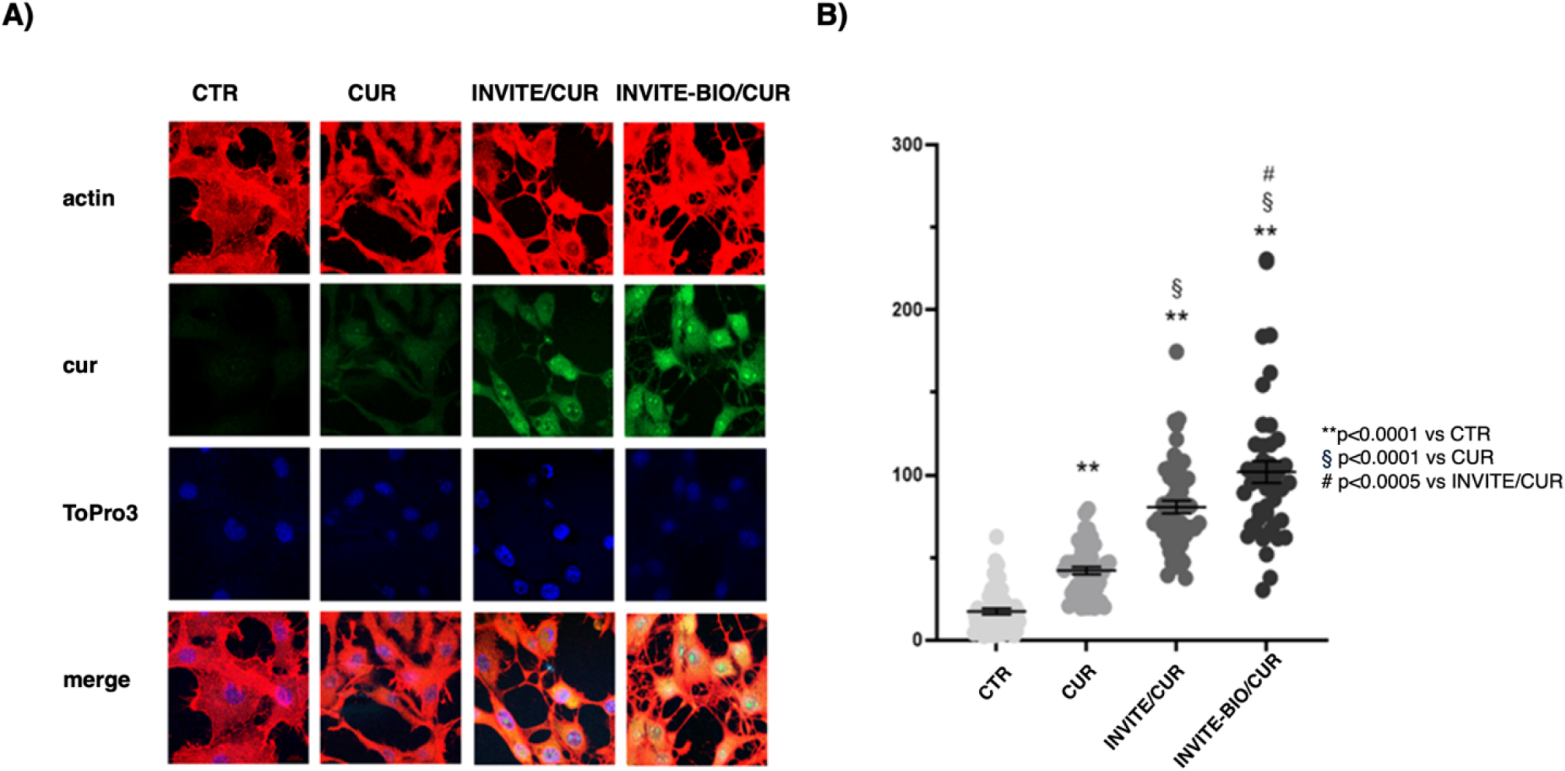
Confocal analysis of curcumin internalization in U87MG cells. (A) Representative confocal images of U87MG cells treated with vehicle (CTR), 10 μg/mL curcumin (CUR), curcumin-loaded INVITE micelles (INVITE/CUR), and curcumin-loaded INVITE-BIO micelles (INVITE-BIO/CUR). Actin staining is visualized in red, curcumin in green, and nuclei in blue. Scale bar = 10 μm. (B) Graphic representation of curcumin fluorescence intensity analysis on U87MG cells, treated as above-reported, from confocal microscope images. ** *p* < 0.0001 vs CTR, § *p* < 0.0001 vs CUR, # *p* < 0.0005 vs INVITE/CUR, one-way ANOVA followed by Tukey’s multiple comparison test.

Microscopic analysis (confocal microscopy) revealed a slightly higher fluorescence intensity for the biotinylated nanosystems, suggesting enhanced cellular internalization. Consequently, to further investigate the dynamics of the interaction between the micelles and tumor cells, we performed additional studies using flow cytometry.

U87MG cells were treated with the micelles and analyzed by flow cytometry, and data revealed that both nanosystems INVITE/CUR and INVITE-BIO/CUR were internalized by tumor cells, but the internalization of the biotin-functionalized carriers is faster than INVITE micelles, as shown by a greater number of fluorescent cells and higher fluorescence intensity after incubation times of 1, 3, and 6 h (Figure 7A and B; Figure S4A). Notably, the uptake of biotinylated nanosystems increased progressively over time, whereas the nonbiotinylated carriers showed a slight decrease in fluorescent cell numbers (Figure 7B), suggesting possible efflux of the lNVITE systems.

**Figure 7:**
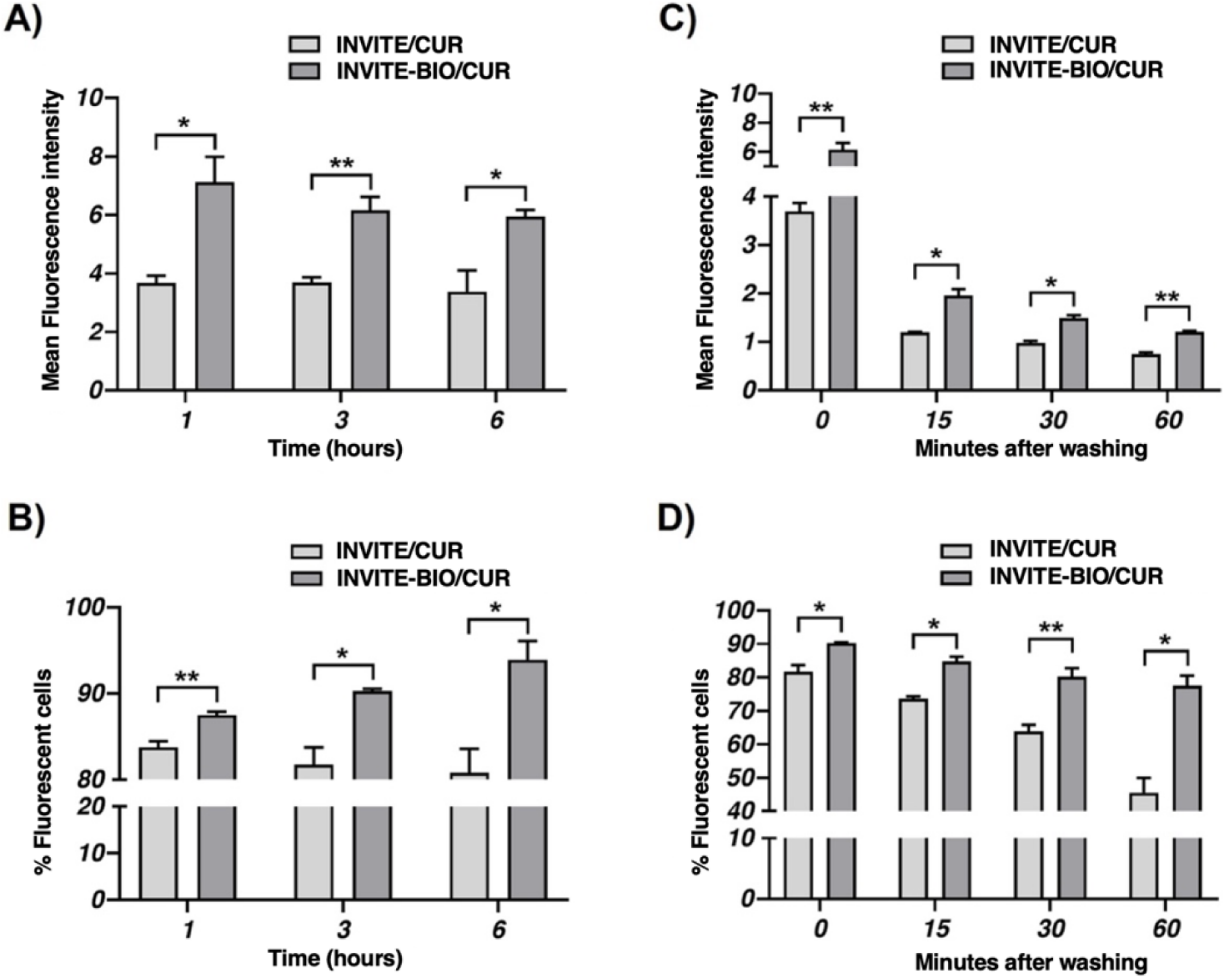
Cellular uptake and retention studies of INVITE/CUR and INVITE-BIO/CUR micelles at 30 μM curcumin concentration as from flow cytofluorimeter analysis: (A) mean fluorescence intensity after incubation periods of 1, 3, and 6 h; (B) number (%) of fluorescent cells after incubation periods of 1, 3, and 6 h; (C) mean fluorescence intensity after an incubation period of 3 h and washing at 0, 15, 30, and 60 min; (D) number (%) of fluorescent cells after an incubation period of 3 h and washing at 0, 15, 30, and 60 min. Data are presented as mean ± SEM. Statistical significance was assessed using Student’s *t*-test. *p < 0.05, **p < 0.01.

Further, when tumor cells were incubated with the nanocarriers for 3 h and then washed to remove the extracellular content of micelles, we observed that the number of fluorescent cells remained relatively constant for the INVITE-BIO/CUR, indicating a superior cellular retention (Figure 7D; Figure S4A). Indeed, although fluorescence intensity decreased over time postwashing, it remained consistently higher than in cells treated with nonbiotinylated carriers (INVITE/CUR) (Figure 7C; Figure S4A), suggesting a sustained intracellular presence despite a potential release and degradation of the curcumin.

A receptor competition assay was performed to confirm the involvement of biotin receptors in the cellular uptake of the developed nanosystems. U87 cells were incubated for 3 h with INVITE/CUR and INVITE-BIO/CUR nanosystems, where curcumin is used as a fluorescent probe to monitor cell-associated fluorescence. The percentage of fluorescent cells was quantified in the absence and presence of a 10-fold excess of free biotin. As from Figure 8, for the INVITE/CUR micelles, the coincubation with free biotin did not show significant changes in the percentage of fluorescent cells, suggesting that their cellular uptake is not influenced by receptors blocking and is likely attributable to nonspecific uptake mechanisms.

**Figure 8:**
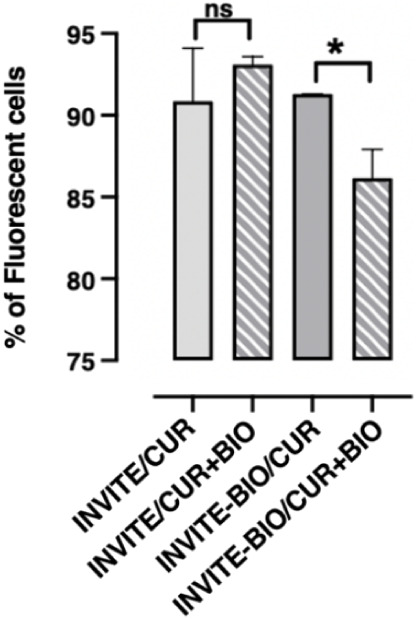
*In vitro* receptor competition assay evaluating the effect of free biotin on INVITE/CUR and INVITE-BIO/CUR micelles. Number (%) of fluorescent cells after an incubation period of 3 h in the condition of INVITE/CUR and INVITE-BIO/CUR nanosystems, measured in the presence or absence of a 10-fold excess of free biotin. Data are presented as mean ± SEM. Statistical significance was assessed using Student’s *t*-test. *p < 0.05, ns = not significant.

In the case of INVITE-BIO/CUR micelles, the presence of free biotin led to a statistically significant reduction in the percentage of fluorescent cells. These data are consistent with a competitive effect at the receptor level and suggest the involvement of a biotin receptor-mediated uptake.

Collectively, *in vitro* biological findings indicate that the presence of biotin moieties enhances the cellular uptake and retention of the INVITE nanosystem, which may translate to improved intracellular drug delivery *in vivo.*

To better understand the fate of the INVITE-BIO micelles after their i.v. administration and to assess the ability of systems to overcome the BBB, a preliminary *in vivo* evaluation was performed on a zebrafish (*Danio rerio*) model that, thanks to its optical transparency and the evolutionary conservation of major physiological pathways, is suitable for real-time evaluation of nanoparticle trafficking and tissue accumulation.^37–39^

The toxicity assays performed in zebrafish embryos at a CUR concentration of 10 μM (Figure 9) revealed that exposure to the free drug resulted in evident toxic effects at 48 hpf with 20% survival, consistent with its intrinsic bioactivity and its unrestricted interaction with biological membranes and developmental pathways.^40–42^ In contrast, INVITE/CUR and INVITE-BIO/CUR do not elicit any detectable adverse effects, suggesting that nanoencapsulation can effectively attenuate the early-stage toxicity of the drug *in vivo* and that micelles not only improve the solubility of the drug, allowing its i.v. administration, but also enhance the safety profile, modulating the interaction between the drug and the biological environment.

**Figure 9:**
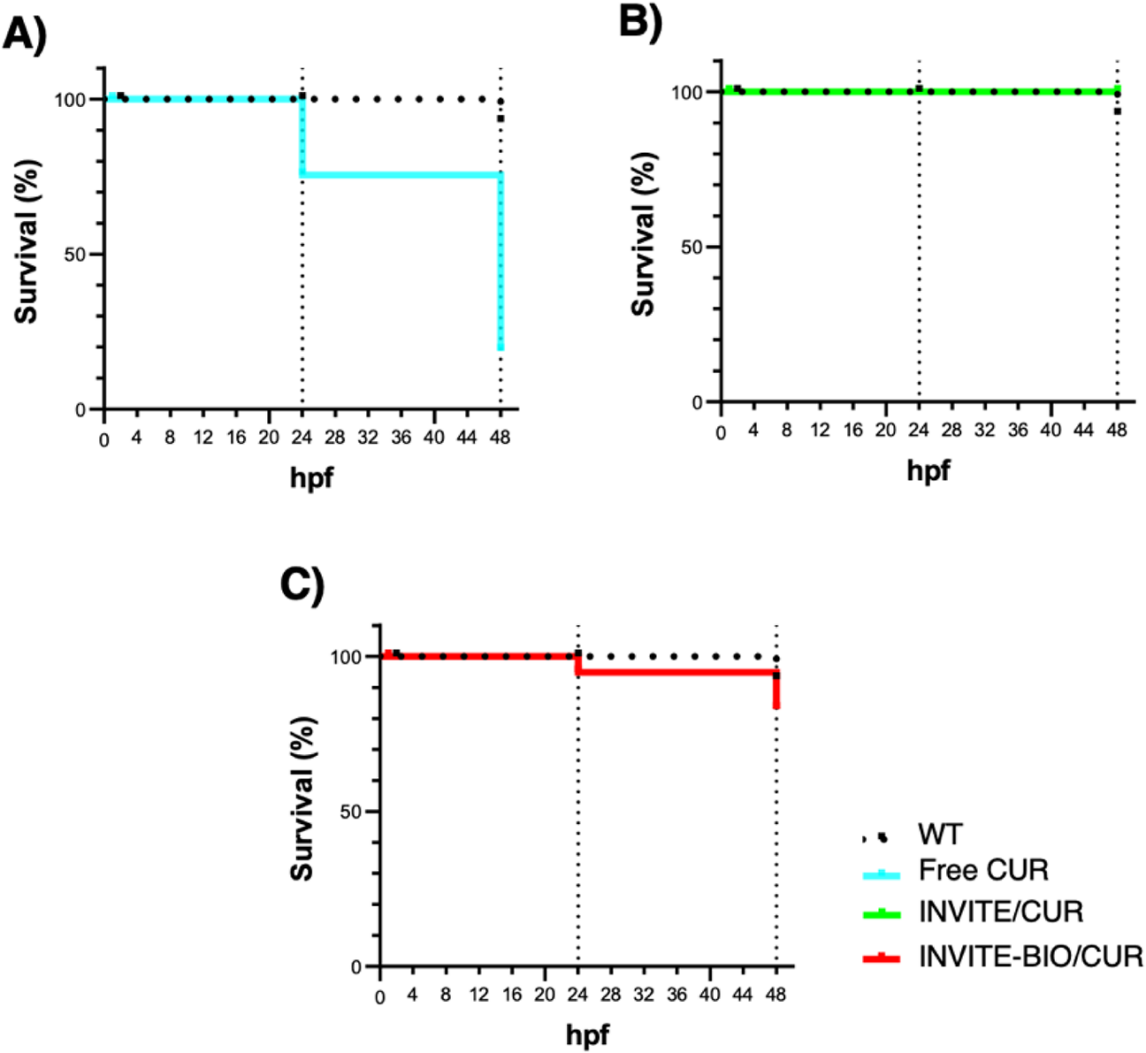
Quantification of WT zebrafish survival. Embryos at 2-cell stage were exposed to 10 μM of free CUR (A), INVITE/CUR (B), and INVITE-BIO/CUR (C). Samples are represented as follows: untreated WT as black dotted line, WT treated with free CUR as light blue line, WT treated with INVITE/CUR as green line, and INVITE-BIO/CUR as red line. 39 WT, 33 free CUR-treated, 27 INVITE/CUR-treated, and 22 INVITE-BIO/CUR embryos were analyzed. The experiment was repeated in triplicate.

Biodistribution in zebrafish clearly demonstrates the pivotal role of the biotin in enhancing the cerebral localization of the INVITE micelles. As shown in Figure 10, the significantly higher curcumin fluorescent intensity at the cerebral level of INVITE-BIO/CUR micelles indicates a pronounced accumulation in the brain region compared to the nontargeted formulations. This suggests enhanced recognition, binding, and receptor-mediated internalization mechanisms, collectively supporting a more selective uptake by neural tissues and, ultimately, facilitating improved translocation across physiological barriers analogous to the BBB in zebrafish.

**Figure 10:**
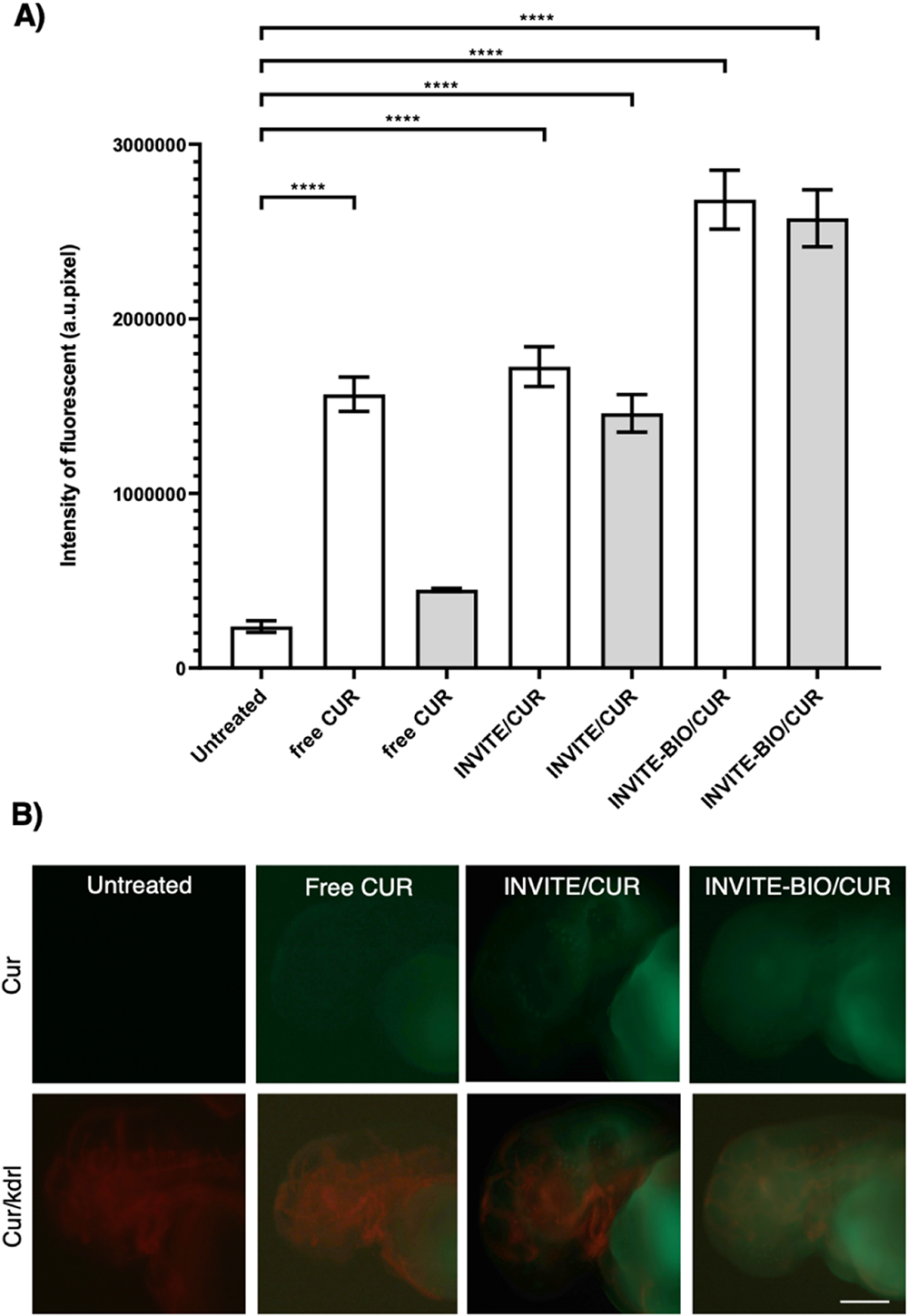
Compounds’ bioavailability and biodistribution in zebrafish. Quantification of fluorescence intensity level after treatment via injection at 48 hpf. (A) Curcumin fluorescence intensity was measured at the cerebral level in zebrafish embryos after injection, in the subintestinal vein plexus site level, at 2 mM of free CUR, INVITE/CUR, and INVITE-BIO/CUR. The bars in the graph report the raw data concerning the measurement taken shortly after injection (48 hpf) (white bars) and data of samples taken 24 h post injection (72 hpf) (gray bars). Numbers of analyzed samples: untreated WT (*n* = 74), free CUR shortly after injection (*n* = 48), free CUR 24 h post injection (*n* = 47), INVITE/CUR shortly after injection (*n* = 93), INVITE/CUR 24 h post injection (*n* = 84), INVITE-BIO/CUR samples shortly after injection (*n* = 65), and INVITE-BIO/CUR samples 24 h post injection (*n* = 65). The experiment was repeated in triplicate. The graphs report the quantification of green fluorescence signal as the mean of raw integrated density (number of fluorescent pixels) ± Std Error. Error bars are reported for all analyzed conditions. ** *p* < 0.01, **** *p* < 0.0001 according to one-way ANOVA with Bonferroni correction; all outliers were removed from the analysis. (B) Representative images of curcumin-positive zebrafish at 72 hpf, after injection (4 nL of 2 mM free CUR, INVITE/CUR, and INVITE-BIO/CUR) and the respective merged images showing in green curcumin presence and in red the Kdrl:Mcherry reporter line. Scale bar, 200 μM.

In addition, from the representative images (Figure 10B) it is possible to note a homogeneous spatial distribution of INVITE-BIO/CUR micelles within the cerebral region that can be useful to reach the infiltrating tumor cells that frequently escape from the GBM primary tumor mass. Thus, we can postulate that this widespread dispersion increases the chance of reaching these migratory, treatment-resistant cells responsible for recurrence and poor prognosis.

To the best of our knowledge, compared with other nanosystems,^43–45^ this study represents the first report of a fully natural polysaccharide–vitamin E micellar carrier specifically designed for glioblastoma therapy. In fact, the INVITE-BIO micelles are entirely composed of the natural substances inulin and vitamin E and functionalized with biotin, an endogenous vitamin, previously investigated as a glioblastoma-targeting ligand but never incorporated into a micellar architecture. Importantly, the exclusive use of naturally derived building blocks relies on materials obtained from renewable sources and avoids the use of synthetic polymers. Moreover, INVITE-BIO micelles exhibited efficient drug loading with controlled release and enhanced dual-targeting toward both the blood–brain barrier and glioblastoma cells. Overall, these elements highlight the originality of INVITE-BIO micelles as a natural, dual-targeted platform for the delivery of lipophilic antitumor agents in glioblastoma treatment.

## 4 Conclusions

In this work, for the first time, we demonstrated that INVITE-BIO nanomicelles exhibit a dual-targeting ability, thanks to the surface-exposed biotin moieties.

*In vitro* biological characterizations on U87MG GBM cells and an *in vivo* biodistribution study in a zebrafish model collectively revealed the critical role of the biotin surface ligand to help the INVITE systems overcome the BBB, thereby increasing the intracerebral localization, and to selectively target the GBM cells, where they are rapidly internalized and retained.

Moreover, INVITE-BIO nanomicelles efficiently encapsulate and release the hydrophobic drug curcumin in a controlled manner, confirming their suitability as carriers for lipophilic anticancer agents. A key advantage of this system over conventional polymeric nanosystems lies in its entirely natural composition based on inulin, vitamin E, and biotin, which contributes to its biocompatibility and potential translational relevance.

The experimental evidence strongly supports our hypothesis that targeted functionalization represents an effective strategy to improve the cerebral tropism of drug delivery systems, with clear implications for the treatment of brain tumors requiring precise and efficient brain delivery.

Therefore, it can be concluded that intravenously administered, long-circulating curcumin-loaded biotinylated INVITE polymeric micelles (INVITE-BIO/CUR) represent a very promising dual-targeting Drug Delivery System and can be proposed as a nanocarrier for targeted delivery of lipophilic antitumor drugs in the treatment of tumors expressing the BIO receptors, such as glioblastoma multiforme.

Future studies will be addressed to explore the INVITE-BIO nanosystems in more complex biological models to corroborate the potential therapeutic advantages conferred by biotin targeting.

## Supplementary Material



## Data Availability

The data supporting this article have been included as part of the ESI.

## Notes

Additional experimental details, figures, tables, and characterization data, including the plot of the I_1_/I_3_ ratio of the hydrophobic probe pyrene vs INVITE-BIO concentrations (Figure S1); additional ^1^H NMR spectrum of INVITE conjugate (Figure S2) and INVITE-BIO conjugate (Figure S3); and cytofluorimetric evaluation of curcumin fluorescence dynamics in U87 cells following INVITE-curcumin and INVITE-biocurcumin treatment (Figure S4) (PDF)

## Notes

This work is dedicated to Professor Giuseppe Tripodo.

## References

[1] Iturrioz-Rodríguez N., Bertorelli R., Ciofani G. (2021). Lipid-Based Nanocarriers for The Treatment of Glioblastoma. Adv. NanoBiomed Res..

[2] Wu W., Klockow J. L., Zhang M., Lafortune F., Chang E., Jin L., Wu Y., Daldrup-Link H. E. (2021). Glioblastoma Multiforme (GBM): An Overview of Current Therapies and Mechanisms of Resistance. Pharmacol. Res..

[3] Mittal S., Ali J., Baboota S. (2021). Overcoming the Challenges in the Treatment of Glioblastoma via Nanocarrier- Based Drug Delivery Approach. Curr. Pharm. Des..

[4] Ayub A., Wettig S. (2022). An Overview of Nanotechnologies for Drug Delivery to the Brain. Pharmaceutics.

[5] Jena L., McErlean E., McCarthy H. (2020). Delivery across the Blood-Brain Barrier: Nanomedicine for Glioblastoma Multiforme. Drug Delivery Transl. Res..

[6] Riccobelli P., Pierce B. F., Craparo E. F., Bonini S. A., Pasut G., Fanzani A., Mandracchia D. (2025). Advances in Nanoparticle Systems for Targeted Therapy against Glioblastoma Multiforme. Int. J. Pharm..

[7] Qamar Z., Sartaj A., Iqubal M. K., Qizilbash F. F., Sabir S., Ali J., Ali A., Baboota S. (2023). Combination Drug Loaded Lipid-Based Nanocarriers as Treatment Entity for Battling Glioblastoma Multiforme. J. Drug Delivery Sci. Technol..

[8] Rodà F., Caraffi R., Picciolini S., Tosi G., Vandelli M. A., Ruozi B., Bedoni M., Ottonelli I., Duskey J. T. (2023). Recent Advances on Surface-Modified GBM Targeted Nanoparticles: Targeting Strategies and Surface Characterization. Int. J. Mol. Sci..

[9] Reddy S., Tatiparti K., Sau S., Iyer A. K. (2021). Recent Advances in Nano Delivery Systems for Blood-Brain Barrier (BBB) Penetration and Targeting of Brain Tumors. Drug Discovery Today.

[10] Tripodo G., Mandracchia D., Collina S., Rui M., Rossi D. (2014). New Perspectives in Cancer Therapy: The Biotin-Antitumor Molecule Conjugates. Med. Chem..

[11] Lai S. W., Lin H. J., Liu Y. S., Yang L. Y., Lu D. Y. (2020). Monocarboxylate Transporter 4 Regulates Glioblastoma Motility and Monocyte Binding Ability. Cancers.

[12] Miranda-Gonçalves V., Gonçalves C. S., Granja S., de Castro J. V., Reis R. M., Costa B. M., Baltazar F. (2021). MCT1 Is a New Prognostic Biomarker and Its Therapeutic Inhibition Boosts Response to Temozolomide in Human Glioblastoma. Cancers.

[13] Uram Ł., Misiorek M., Pichla M., Filipowicz-Rachwał A., Markowicz J., Wołowiec S., Wałajtys-Rode E. (2019). The Effect of Biotinylated PAMAM G3 Dendrimers Conjugated with COX-2 Inhibitor (Celecoxib) and PPARγ Agonist (Fmoc-L-Leucine) on Human Normal Fibroblasts, Immortalized Keratinocytes and Glioma Cells in Vitro. Molecules.

[14] Uchida Y., Ito K., Ohtsuki S., Kubo Y., Suzuki T., Terasaki T. (2015). Major Involvement of Na ± Dependent Multivitamin Transporter (SLC5A6/SMVT) in Uptake of Biotin and Pantothenic Acid by Human Brain Capillary Endothelial Cells. J. Neurochem..

[15] Akhter H., Rizwanullah, Ahmad J., Amin S., Ahmad M. Z., Minhaj A., Mujtaba A., Ali J. (2021). Molecular Targets and Nanoparticulate Systems Designed for the Improved Therapeutic Intervention in Glioblastoma Multiforme. Drug Res..

[16] Yoon J., Grinchuk O. V., Kannan S., Ang M. J. Y., Li Z., Tay E. X. Y., Lok K. Z., Lee B. W. L., Chuah Y. H., Chia K. (2021). A Chemical Biology Approach Reveals a Dependency of Glioblastoma on Biotin Distribution. Sci. Adv..

[17] Neves A. R., Queiroz J. F., Weksler B., Romero I. A., Couraud P.-O., Reis S. (2015). Solid Lipid Nanoparticles as a Vehicle for Brain-Targeted Drug Delivery: Two New Strategies of Functionalization with Apolipoprotein E. Nanotechnology.

[18] Mandracchia D., Tripodo G., Latrofa A., Dorati R. (2014). Amphiphilic Inulin-d-α-Tocopherol Succinate (INVITE) Bioconjugates for Biomedical Applications. Carbohydr. Polym..

[19] Mandracchia D., Trapani A., Chlapanidas T., Trapani G., Tripodo G. (2015). Enzyme Controlled Release of Celecoxib from Inulin Based Nanomicelles. J. Cell. Biotechnol..

[20] Tripodo G., Chlapanidas T., Perteghella S., Vigani B., Mandracchia D., Trapani A., Galuzzi M., Tosca M. C., Antonioli B., Gaetani P., Marazzi M., Torre M. L. (2015). Mesenchymal Stromal Cells Loading Curcumin-INVITE-Micelles: A Drug Delivery System for Neurodegenerative Diseases. Colloids Surf., B.

[21] Tripodo G., Pasut G., Trapani A., Mero A., Lasorsa F. M., Chlapanidas T., Trapani G., Mandracchia D. (2015). Inulin- d -α-Tocopherol Succinate (INVITE) Nanomicelles as a Platform for Effective Intravenous Administration of Curcumin. Biomacromolecules.

[22] Mandracchia D., Tripodo G., Trapani A., Ruggieri S., Annese T., Chlapanidas T., Trapani G., Ribatti D. (2016). Inulin Based Micelles Loaded with Curcumin or Celecoxib with Effective Anti-Angiogenic Activity. Eur. J. Pharm. Sci..

[23] Mandracchia D., Rosato A., Trapani A., Chlapanidas T., Montagner I. M., Perteghella S., Di Franco C., Torre M. L., Trapani G., Tripodo G. (2017). Design, Synthesis and Evaluation of Biotin Decorated Inulin-Based Polymeric Micelles as Long-Circulating Nanocarriers for Targeted Drug Delivery. Nanomedicine.

[24] Baliyan D., Sharma R., Goyal S., Chhabra R., Singh B. (2025). Phytochemical Strategies in Glioblastoma Therapy: Mechanisms, Efficacy, and Future Perspectives. Biochim. Biophys. Acta, Mol. Basis Dis..

[25] Walker B. C., Mittal S. (2020). Antitumor Activity of Curcumin in Glioblastoma. Int. J. Mol. Sci..

[26] Hesari A., Rezaei M., Rezaei M., Dashtiahangar M., Fathi M., Rad J. G., Momeni F., Avan A., Ghasemi F. (2019). Effect of Curcumin on Glioblastoma Cells. J. Cell. Physiol..

[27] Brandes A. A., Bartolotti M., Tosoni A., Franceschi E. (2016). Nitrosoureas in the Management of Malignant Gliomas. Curr. Neurol. Neurosci. Rep..

[28] Ambele M. A., Maebele L. T., Mulaudzi T. V., Kungoane T., Damane B. P. (2024). Advances in Nano-Delivery of Phytochemicals for Glioblastoma Treatment. Discover Nano.

[29] Tripodo G., Mandracchia D., Dorati R., Latrofa A., Genta I., Conti B. (2013). Nanostructured Polymeric Functional Micelles for Drug Delivery Applications. Macromol. Symp..

[30] de Freitas C. F., Montanha M. C., Pellosi D. S., Kimura E., Caetano W., Hioka N. (2019). Biotin-Targeted Mixed Liposomes: A Smart Strategy for Selective Release of a Photosensitizer Agent in Cancer Cells. Mater. Sci. Eng..

[31] Priyadarsini K. I. (2009). Photophysics Photochemistry and Photobiology of Curcumin: Studies from Organic Solutions, Bio-Mimetics and Living Cells. J. Photochem. Photobiol., C.

[32] Craig M. P., Gilday S. D., Dabiri D., Hove J. R. (2012). An Optimized Method for Delivering Flow Tracer Particles to Intravital Fluid Environments in the Developing Zebrafish. Zebrafish.

[33] Counil H., Silva R. O., Rabanel J.-M., Zaouter C., Haddad M., Khedher M. R. B., Brambilla D., Fülöp T., Patten S. A., Ramassamy C. (2025). Brain Penetration of Peripheral Extracellular Vesicles from Alzheimer’s Patients and Induction of Microglia Activation. J. Extracell. Biol..

[34] Catenacci L., Mandracchia D., Sorrenti M., Colombo L., Serra M., Tripodo G. (2014). In-Solution Structural Considerations by ^1^ H NMR and Solid-State Thermal Properties of Inulin-dB1;-Tocopherol Succinate (INVITE) Micelles as Drug Delivery Systems for Hydrophobic Drugs. Macromol. Chem. Phys..

[35] Malec K., Monaco S., Delso I., Nestorowicz J., Kozakiewicz-Latała M., Karolewicz B., Khimyak Y. Z., Angulo J., Nartowski K. P. (2023). Unravelling the Mechanisms of Drugs Partitioning Phenomena in Micellar Systems via NMR Spectroscopy. J. Colloid Interface Sci..

[36] Nakamura H., Watano S. (2018). Direct Permeation of Nanoparticles across Cell Membrane: A Review. KONA Powder Part. J..

[37] Cassar S., Adatto I., Freeman J. L., Gamse J. T., Iturria I., Lawrence C., Muriana A., Peterson R. T., Van Cruchten S., Zon L. I. (2020). Use of Zebrafish in Drug Discovery Toxicology. Chem. Res. Toxicol..

[38] Chaoul V., Dib E.-Y., Bedran J., Khoury C., Shmoury O., Harb F., Soueid J. (2023). Assessing Drug Administration Techniques in Zebrafish Models of Neurological Disease. Int. J. Mol. Sci..

[39] Nagdiya D., Arora S., Kumar V., Kumar D., Singh A., Singh C. (2025). Application of Casein Micelles for Targeting Huntington’s Disease in Experimental Zebrafish Model. Mol. Neurobiol..

[40] Pucci G., Savoca G., Iacoviello G., Russo G., Forte G. I., Cavalieri V. (2024). Curcumin’s Radioprotective Effects on Zebrafish Embryos. Antioxidants.

[41] Wu J.-Y., Lin C.-Y., Lin T.-W., Ken C.-F., Wen Y.-D. (2007). Curcumin Affects Development of Zebrafish Embryo. Biol. Pharm. Bull..

[42] Igartúa D. E., Martinez C. S., Alonso S. D. V., Chiaramoni N. S., Prieto M. J. (2020). Toxicity Assessment of Free and Dendrimer-Complexed Curcumin in Zebrafish Larvae. PharmaNutrition.

[43] Şahin Ş., Kaya-Tilki E., Baysal M., Öztürk A. A. (2025). Preparation and Evaluation of Temozolomide Loaded PLGA Nanoparticles for the Treatment of Glioblastoma Multiforme. Sci. Rep..

[44] Pashirova T. N., Nemtarev A. V., Buzyurova D. N., Shaihutdinova Z. M., Dimukhametov M. N., Babaev V. M., Voloshina A. D., Mironov V. F. (2024). Terpenes-Modified Lipid Nanosystems for Temozolomide, Improving Cytotoxicity against Glioblastoma Human Cancer Cells In Vitro. Nanomaterials.

[45] Luiz M. T., Delello Di Filippo L., Tofani L. B., de Araújo J. T. C., Dutra J. A. P., Marchetti J. M., Chorilli M. (2021). Highlights in Targeted Nanoparticles as a Delivery Strategy for Glioma Treatment. Int. J. Pharm..

