# Supplementary material for: Biotin-Decorated Inulin-Based Polymeric Micelles Unveil Their Dual-Targeting Ability for the Potential Treatment of Glioblastoma Multiforme through the In Vitro and In Vivo Investigations
Source: Mol Pharm. 2026 Apr 13;23(5):3009–22. doi: 10.1021/acs.molpharmaceut.5c01861 (PMC13155663; doi:10.1021/acs.molpharmaceut.5c01861)
Supplement: Supplementary file 1 [file mp5c01861_si_001.pdf]

Supporting Information

Biotin-decorated inulin-based polymeric micelles unveil their dual-targeting ability for the potential treatment of glioblastoma multiforme through the *in vitro* and *in vivo* investigations

Paola Riccobelli[1], Elena Cannone[1], Serena Filiberti[1], Giovanni Ribaudo[1], Sara Anna Bonini[1], Antonella Grigoletto[2], Maria Luisa Massardi[1], Silvia Codenotti[1], Marco Schiavone[1], Roberto Ronca[1], Delia Mandracchia[1]*

[1]Department of Molecular and Translational Medicine, University of Brescia, Viale Europa 11, 25123 Brescia, Italy.

[2]Department of Pharmaceutical and Pharmacological Sciences, University of Padova, Via Marzolo 5, Padova 35131, Italy.

Corresponding author:

Delia Mandracchia, Department of Molecular and Translational Medicine, University of Brescia, Brescia 25123, ITALY.

Email: delia.mandracchia@unibs.it

ORCID: 0000-0002-7101-013X

Supporting info 1

Determination of INVITE-BIO Critical Association Concentration (CAC)

The Critical Association Concentration (CAC) of INVITE-BIO in ultrapure water was determined by fluorescence spectroscopy using pyrene. The method proposed by Kalyanasundaram et al. was applied by considering the change in the vibronic fine structure of the pyrene emission and by monitoring the changes in the ratio of the intensities I1 and I3 of the first and third vibronic peaks [1-4]. Excitation was done at 334 nm and emission were recorded in the 350-450 nm wavelength range. Pyrene was diluted in the INVITE-BIO samples solutions to yield a concentration of $2 \cdot 10^{-6}$ M. In particular, a stock solution of pyrene in acetone (6×10^{-3} M) was prepared, then, a small amount of this pyrene stock solution was diluted with water to obtain a final concentration of pyrene $2 \cdot 10^{-5}$ M. This pyrene water solution was maintained under vigorous stirring in order to totally remove the acetone.

A proper amount of the pyrene solution was added to the INVITE-BIO solutions at various concentrations in the range of 10^{-4} -2.5 mg/ml and was allowed to stand overnight to equilibrate. Fluorescence intensities of the pyrene entrapped in the micelle core were determined at room temperature and the ratio of the intensities I1 and I3 against INVITE-BIO concentration was plotted (Figure S1).

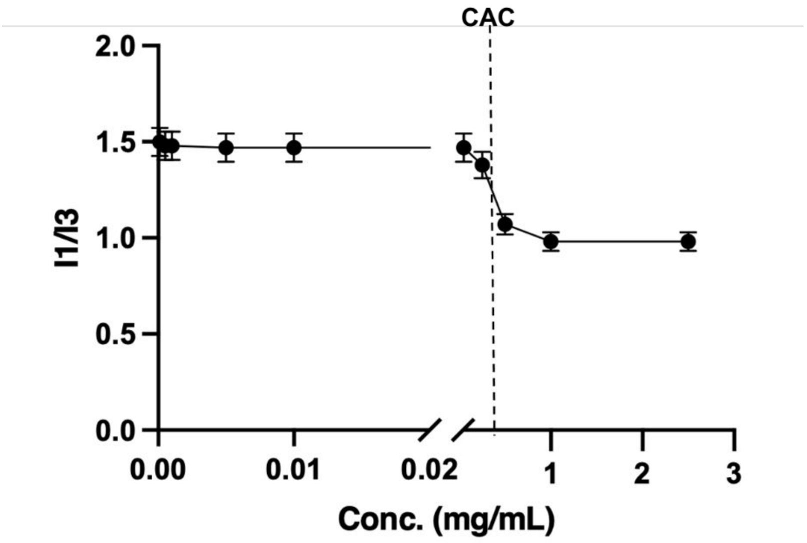

Figure S1. Plot of the I_1/I_3 ratio of the hydrophobic probe pyrene (determined by fluorescence spectroscopy) *vs* INVITE-BIO concentrations.

[1] D. Mandracchia, G. Tripodo, A. Latrofa, R. Dorati, Amphiphilic inulin-d-alpha-tocopherol succinate (INVITE) bioconjugates for biomedical applications, Carbohydrate polymers, 103 (2014) 46-54.

[2] L. Catenacci, D. Mandracchia, M. Sorrenti, L. Colombo, M. Serra, G. Tripodo, In-Solution Structural Considerations by H-1 NMR and Solid-State Thermal Properties of Inulin-D-alpha-Tocopherol Succinate (INVITE) Micelles as Drug Delivery Systems for Hydrophobic Drugs, Macromolecular Chemistry and Physics, 215 (2014) 2084-2096.

[3] A. Spura, R.U. Riel, N.D. Freedman, S. Agrawal, C. Seto, E. Hawrot, Biotinylation of substituted cysteines in the nicotinic acetylcholine receptor reveals distinct binding modes for alpha-bungarotoxin and erabutoxin a, Journal of Biological Chemistry, 275 (2000) 22452-22460.

[4] K. Kalyanasundaram, J.K. Thomas, Environmental effects on vibronic band intensities in pyrene monomer fluorescence and their application in studies of micellar systems, Journal of the American Chemical Society, 99 (1977) 2039-2044.

Supporting info 2

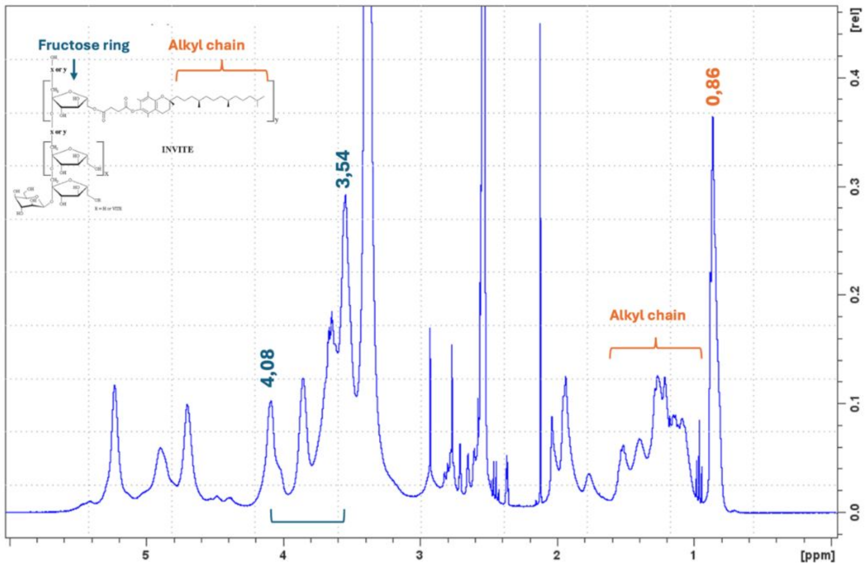

Figure S2. Representative ¹H–NMR spectrum of INVITE conjugate recorded in DMSO-d_6. The figure highlights the peaks at 0.86 ppm (12H VITE) and 3.50-4.00 ppm (7H INU), which are characteristic of the INVITE conjugate.

<u>**Supporting info 3**</u>

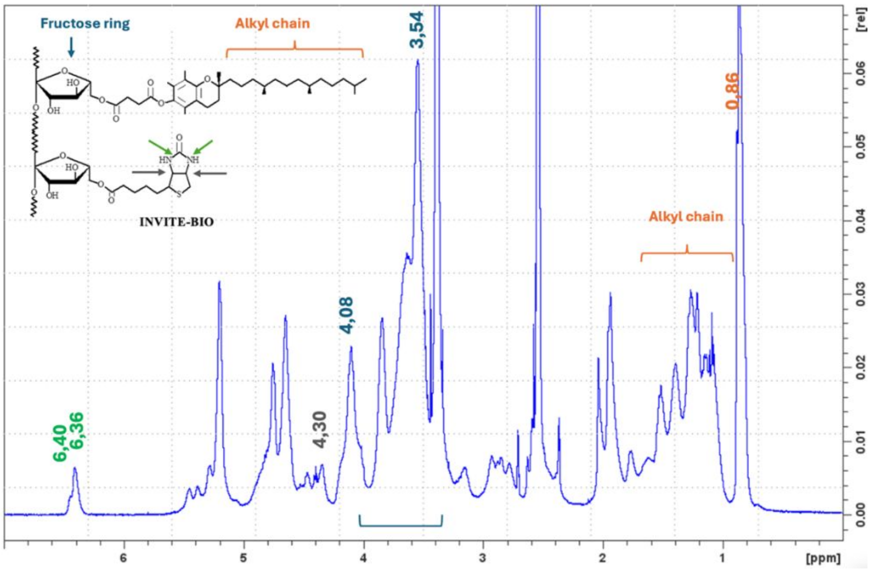

Figure S3. Representative ¹H–NMR spectrum of INVITE-BIO conjugate recorded in DMSO-d_6. The figure highlights the peaks at 0.86 ppm (12H VITE), 3.50-4.00 ppm (7H INU), 4.30 ppm (1H BIO) and 6.36-6.40 ppm (2NH BIO), which are characteristic of the INVITE-BIO conjugate.

Supporting info 4

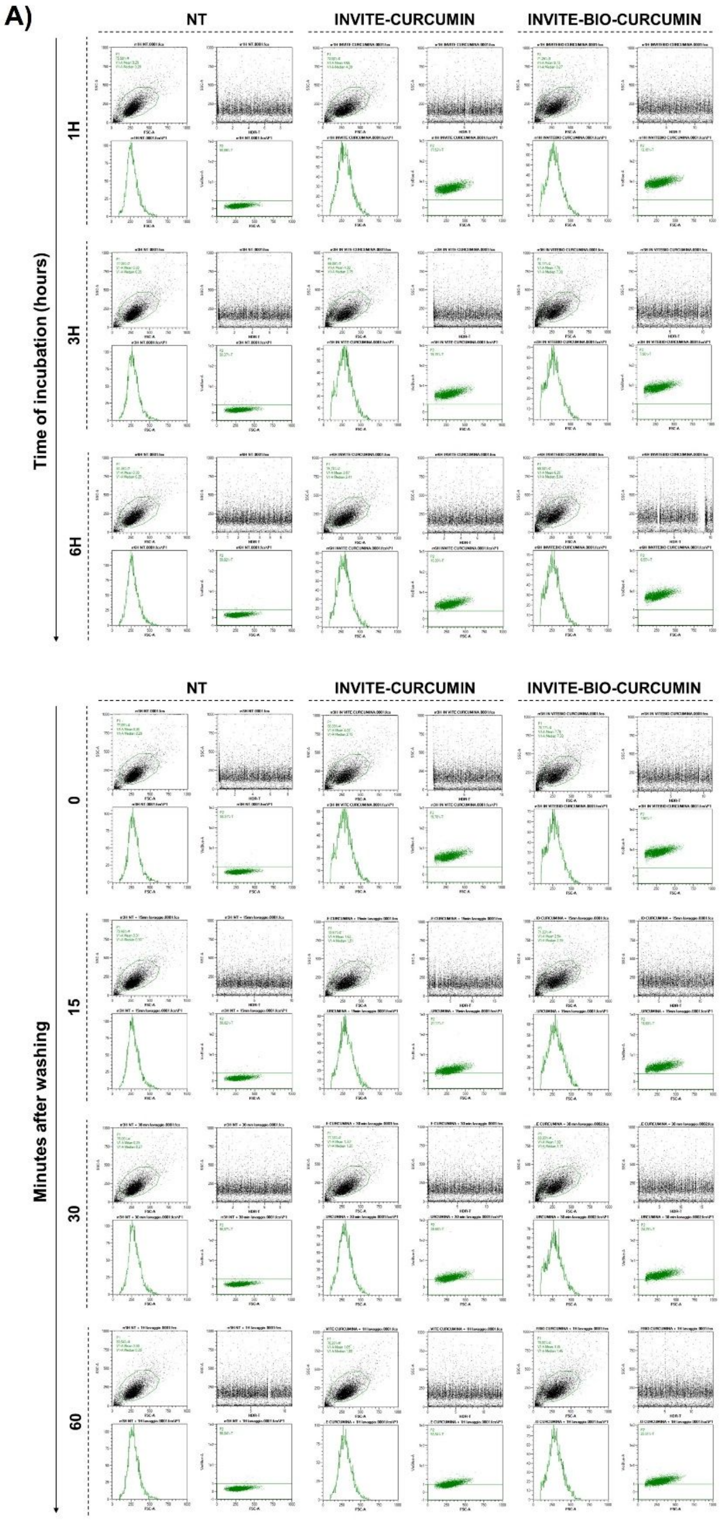

Figure S4. Cytofluorimetric Evaluation of Curcumin Fluorescence Dynamics in U87 Cells Following Invite-curcumin and Invite-Bio-curcumin Treatment: (A) Cytofluorimetric analysis of curcumin fluorescence in U87 cells treated with Invite-curcumin, Invite-Bio-curcumin, or untreated control (NT) for 1, 3, and 6 hours; (B)

Cytofluorimetric analysis of curcumin fluorescence in U87 cells pre-treated with Invite-curcumin, Invite-Bio-curcumin, or control (NT) for 3 hours, followed by washing and analysis at subsequent time points (15, 30, and 60 minutes).
